# Comparison of CEM I and CEM II/A-V in Certyd Lightweight Aggregate Concrete on Bond Strength Development to 15.7 mm Non-Pretensioned Steel Strand

**DOI:** 10.3390/ma19204426

**Published:** 2026-10-21

**Authors:** Andrzej Seruga, Łukasz Ślaga, Rafał Szydłowski, Marcin Midro

**Affiliations:** Department of Civil Engineering, Cracow University of Technology, Warszawska 24 Street, 31-155 Cracow, Poland; andrzej.seruga@pk.edu.pl (A.S.); lukasz.slaga@pk.edu.pl (Ł.Ś.); marcin.midro@pk.edu.pl (M.M.)

**Keywords:** bond stress, embedment length, lightweight aggregate concrete, sintered fly ash artificial aggregate

## Abstract

**Highlights:**

- The concrete–steel strand bond for Portland cement CEM I 52.5R and more ecological fly ash cement CEM II/A-V 52.5 R-NA was investigated.
- The bond of lightweight aggregate concrete to 15.7 mm non-pretensioned steel strands was tested in a pull-out test.
- CEM II/A-V 52.5 R-NA cement, despite its fly ash content, is as well suited for making pretensioned concrete elements as pure Portland cement.

**Abstract:**

The production of pretensioned concrete elements is significantly influenced by the ability to achieve required bond stress values shortly after the element is formed, which is essential for transferring prestressing force. Due to the complex testing procedure, bond strength is not directly measured in practice. Instead, decisions regarding the transfer of prestressing force to the concrete element are based on the tested compressive strength (typically 30–35 MPa). The literature contains studies and guidelines that define bond strength in normal concretes (generally dependent on the compressive strength of the concrete). Such relationships are lacking for lightweight aggregate concretes (LWAC). CEM I 52.5R is the most commonly used type of cement for prestressed concrete elements due to its rapid strength development. However, concrete with CEM II/A-V 52.5R-NA is characterized by better workability and a lower carbon footprint. This paper presents the results of experimental studies on the bond strength of LWAC made with artificial Certyd aggregate and cement CEM II/A-V 52.5R-NA. The obtained results were compared with results from similar previous studies on LWAC using cement CEM I 52.5R. The analysis encompassed bond stress–slip curves and the correlation between bond strength and free-end slip after 3 and 28 days of curing. The tests conducted and the final analysis of the test results pointed out that Certyd lightweight aggregate concrete made with a concrete mixture with cement type CEM I 52.5R, as well as with cement type CEM II/A-V 52.5R-NA, is appropriate for pretensioned concrete members.

## 1. Introduction

The precast concrete sector is presently confronted with the necessity for profound decarbonization, necessitated by the strict climate policies of the European Union and the rising market demand for products with a low carbon footprint [1]. Andrew [2] indicates that the process-related CO_2_ emissions from cement production reached approximately 1.50 ± 0.12 Gt CO_2_ in 2018. Furthermore, life cycle analyses of concrete emphasize that a significant portion of the carbon footprint of concrete arises from the production of cement and clinker [3,4]. Consequently, reducing the clinker factor is currently regarded as one of the most crucial and easily scalable strategies for decarbonizing cementitious materials [3].

The manufacturing of structural elements through prestressed concrete technology is marked by a stringent production regime. A key economic and technological criterion is the achievement of high early strength (often exceeding 30–40 MPa) within a period of less than 24 h. This is a necessary condition for the safe release of the prestressing strands and the rapid removal of tensioning tracks. The conventional and most dependable approach to fulfilling these criteria is the application of pure Portland cement CEM I 52.5R, which is characterized by its rapid setting properties. In this context, it is crucial to compare Portland cement CEM I 52.5 R with siliceous fly ash Portland cement CEM II/A-V 52.5 R-NA. According to the classification in EN 197-1, CEM I consists primarily of 95–100% clinker, whereas CEM II/A-V contains 80–94% clinker and 6–20% siliceous fly ash as its main non-clinker component [5]. Siliceous fly ash is a byproduct generated from the combustion of hard coal in the boilers of power plants. It is captured from flue gases by electrostatic precipitators. This material primarily consists of spherical, glassy particles ranging in size from 1 to 100 microns (similar in size to cement grains). Due to the spherical shape of the particles, it functions like a “ball bearing,” reducing friction, lowering water demand, decreasing the heat of hydration in concrete, and crucially enhancing the workability of lightweight aggregate concrete (LWAC) [6].

Both types of cement can fall under the same strength class of 52.5R, indicating high standard strength and rapid early strength gain. However, their environmental impact differs, as in CEM II/A-V, part of the clinker is replaced with a mineral additive that has a lower assigned emission load.

Literature on life cycle assessment (LCA) consistently indicates that blended cements containing mineral additives, such as fly ash or blast furnace slag, typically exhibit a lower global warming potential (GWP) compared to CEM I [7,8]. Ige and Olanrewaju [8] demonstrated that, in comparison to CEM I, the CEM II/B-V cement, which contains a higher proportion of siliceous fly ash than CEM II/A-V, reduced the GWP index by 29%. This result should not be linearly extrapolated to CEM II/A-V. However, it confirms the prevailing relationship: the lower the clinker content, the smaller the carbon footprint of the cement under similar technological conditions. Similarly, Nath, Sarker, and Biswas [9] showed that replacing 40% of cement with fly ash in high-strength concrete can reduce the carbon footprint and embodied energy while simultaneously enhancing durability in chloride environments.

The environmental benefits of CEM II/A-V extend beyond CO_2_ emissions. The use of fly ash decreases the demand for limestone raw materials and fuels required for clinker firing, while also supporting the utilization of a by-product from coal combustion, thereby reducing the need for its disposal [10]. Research also indicates a positive impact of fly ash on workability, microstructure of the paste, and the concrete’s resistance to chloride ingress and sulfate attack [9,10]. The pozzolanic and filling effects may lead to the sealing of the cement matrix in later stages of curing, which is crucial for durability and indirectly affects the overall environmental impact of the structure. Simultaneously, the evaluation of CEM II/A-V 52.5 R-NA necessitates a multi-criteria approach. The delayed pozzolanic effect of fly ashes at ambient temperature (approximately 20 °C) poses a risk of slowing early strength gain during the initial hours, which directly threatens the fluidity of the production cycle without the application of external heat.

A limited number of experimental investigations have examined the bond stress–slip relationship in lightweight aggregate concrete (LWAC) [11]. Den Uijl [12] conducted experimental investigations on twelve beams measuring 2.94 m in length with cross-sections of 100 × 100 mm and hollow-core slabs manufactured from lightweight aggregate concrete consisting of sintered fly ash (Lytag). The structural elements were pretensioned using two types of steel strands: 9.3 mm and 12.5 mm diameter. The average cubic compressive strength of the concrete at the moment of tensioning force release was 42.0 MPa (f_c,cyl_ = 33.6 MPa) and, after 28 days of maturing, 68.6 MPa (f_c,cyl_ = 54.9 MPa). The research objective was to establish the values of strand slip and transmission length. The average value of transmission lengths determined in the experimental study for strands with a diameter of 12.5 mm ranged from 573 mm to 669 mm for beams and from 543 mm to 673 mm for slabs. Based on the transmission lengths established by the researcher and the reported initial stress values in the strands at the moment of tension release, the authors of this work calculated average bond stresses, which ranged from 3.71 MPa to 4.34 MPa for beams and from 3.55 MPa to 4.40 MPa for slabs. Vázquez-Herrero et al. [13] conducted experimental investigations to determine the bond stress–slip relationship of lightweight aggregate concrete made of Lightweight Expanded Clay Aggregate (LECA, Arlita) to a steel strand of 15.2 mm diameter, employing the methodology developed by Abrishami and Mitchell [14]. The research was performed on cylindrical specimens measuring 300 mm in height and 150 mm in diameter. Although substantial bond stress values ranging from 5 to 6 MPa were achieved at slip values between 0.5 and 3.0 mm, the researchers observed an abrupt failure mechanism during bond loss. The distinct nature of bond deterioration compared to conventional concrete prompted the decision to discontinue further investigations aimed at implementing the production of pretensioned concrete elements manufactured from LECA. Dang, Marti-Vargas, and Hale [15] assert that additional studies are required for pretensioned concrete elements utilizing high-performance concrete and lightweight concrete. Research on LWAC began in Poland with the introduction of a novel artificial aggregate known as Certyd in 2015. This aggregate is manufactured from sintered fly ash. Previous investigations conducted by Seruga et al. [16,17,18] have confirmed the potential for using the artificial lightweight aggregate “Certyd” in the manufacture of pretensioned concrete structures utilizing Portland cement type CEM I 52.5R.

This article presents a comprehensive comparative analysis of the impact of CEM I 52.5 R (concrete type A) and CEM II/A-V 52.5R-NA (concrete type B) on one of the key parameters in concrete utilized for pretensioned concrete components, which is the bond stress. The bond stress directly affects the transmission length and anchorage length of the tendons. Therefore, the correct assessment of bond stress significantly impacts the safety of the entire prestressed concrete structure.

Extensive experimental tests were performed on concrete test specimens for type A concrete, which included both non-pretensioned and tensioned strands. Additionally, the transmission length was measured on beam specimens. The analysis revealed that type A LWAC utilizing CERTYD aggregate is appropriate for manufacturing pretensioned concrete elements [17]. For type B concrete, the initial phase of testing regarding the bond strength of concrete to non-pretensioned strands has been completed.

## 2. Research Aims, Scope and Novelty

The scope of the study is to determine the bond mechanisms and strength of CEM II/A-V cement to lightweight aggregate concrete with Certyd artificial aggregate. This is of great importance in the design of pretensioned concrete elements, given the phasing out of production of CEM I cements with a high carbon footprint. The novelty of this study is the comparison of CEM I and CEM II/A-V cements. The latter has not been studied in terms of bond to prestressing steel.

## 3. Materials and Methods

To establish the bond stress–slip relationships for lightweight aggregate concrete (LWAC) and non-pretensioned plain seven-wire steel strands, experimental investigations were conducted using push-off specimens that were reinforced with an axially embedded steel strand. This experimental research program considered several factors that significantly influence this relationship:

- Bond embedded length (l_emb_ = 40, 80, 120, 240, 330, and 460 mm);
- Concrete compressive strength (for t = 3, 7, and 28 days);
- Type of cement:

Type A—CEM I 52.5 R,

Type B—CEM II/A-V 52.5R-NA.

### 3.1. Test Program

**Figure 1:**
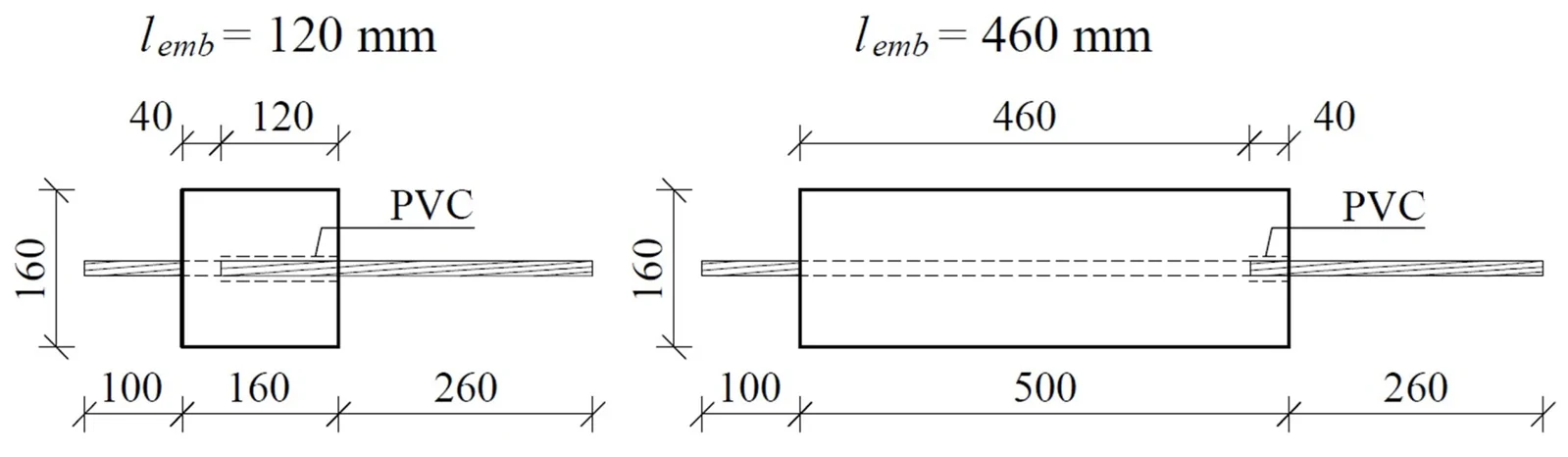
The arrangement of the prestressing steel strand within the individual molds for the test specimens [17].

Research focused on the bond stress between lightweight aggregate concrete and steel prestressing strands was conducted using push-off prism specimens, each measuring 160 mm on all sides, with varying lengths of 160, 280, 370, and 500 mm after curing periods of 3, 7, and 28 days. A total of eight plywood forms for each length were fabricated, resulting in 48 forms specially designed for this study. Prior to the casting process, the strand was secured in a horizontal orientation using rigid protective PVC tubing, with lengths of 120, 80, and 40 mm, in accordance with the specified embedment length. Figure 1 illustrates the configuration of the prestressing steel strand within the separate molds designated for the test specimens.

### 3.2. Concrete

The specimens were constructed using a specially formulated cement concrete mixture, classified as LC 45/50, with the components for 1 m^3^ of the concrete blend detailed in Table 1. For the application of cement CEM I 52.5 R (type A), the concrete specimens were marked as type A specimens. If cement CEM II/A-V 52.5R-NA (type B) was utilized, the concrete samples were designated as type B specimens. These specimens were produced in multiple series, each varying in its active bond length. For each series, standard samples measuring ϕ150 × 300 mm in length were gathered to assess the compressive and tensile strength of the concrete, along with the modulus of elasticity under compression. Following casting, all specimens and standard samples were enveloped in multiple layers of polyethylene sheeting to maintain consistent curing conditions for a duration for the duration of seventy-two hours. Once this time frame elapsed, the specimens were removed from their plywood molds and prepared for experimental evaluation. Experimental investigations were carried out on days 3, 7, and 28 in the case of concrete type A specimens and on days 3 and 28 in the case of concrete type B specimens. After demoulding, all specimens and standard samples intended for testing were kept under three layers of polyethylene sheeting. At each stage of testing, the mechanical properties of the concrete were evaluated according to European standards [19,20,21,22]. The identification numbers of the tested specimens are presented in Table 2 and Table 3.

The compressive strength of both types of concrete, measured after 28 days of curing, was almost identical, showing only a 4% variation. However, after three days of curing, there was a notable 16% difference in compressive strength. The test specimens were created in the laboratory of the Cracow University of Technology, where the ambient temperature ranged from 20 to 22 °C, and the relative humidity was between 40% and 50%. Standard samples for strength evaluation were stored alongside the test specimens, covered with multiple layers of polyethylene sheeting. The significant difference in compressive strength after three days of curing was attributed to the moisture content of the Certyd aggregate, which has a considerable impact on the concrete’s performance.

### 3.3. Steel Prestressing Strands

The bond tests utilized plain seven-wire strands made of steel for prestressing. The strength of the steel was evaluated using a Zwick-Roell Z1200 machine (ZwickRoell GmbH & Co. KG, Ulm, Germany). An integrated force gauge automatically measures the tensile strength. Displacement and strain are recorded by an incremental extensometer that is synchronized with the machine’s software.

The measuring base of the extensometer was 180 mm. The load was applied at a rate of 20 MPa/s until the yield point was reached, after which the control switched automatically to displacement control at a rate of 0.003 1/s within the flow range. The properties of prestressing strands used in experimental investigations are presented in Table 4 and Table 5.

### 3.4. Test Procedure

**Figure 2:**
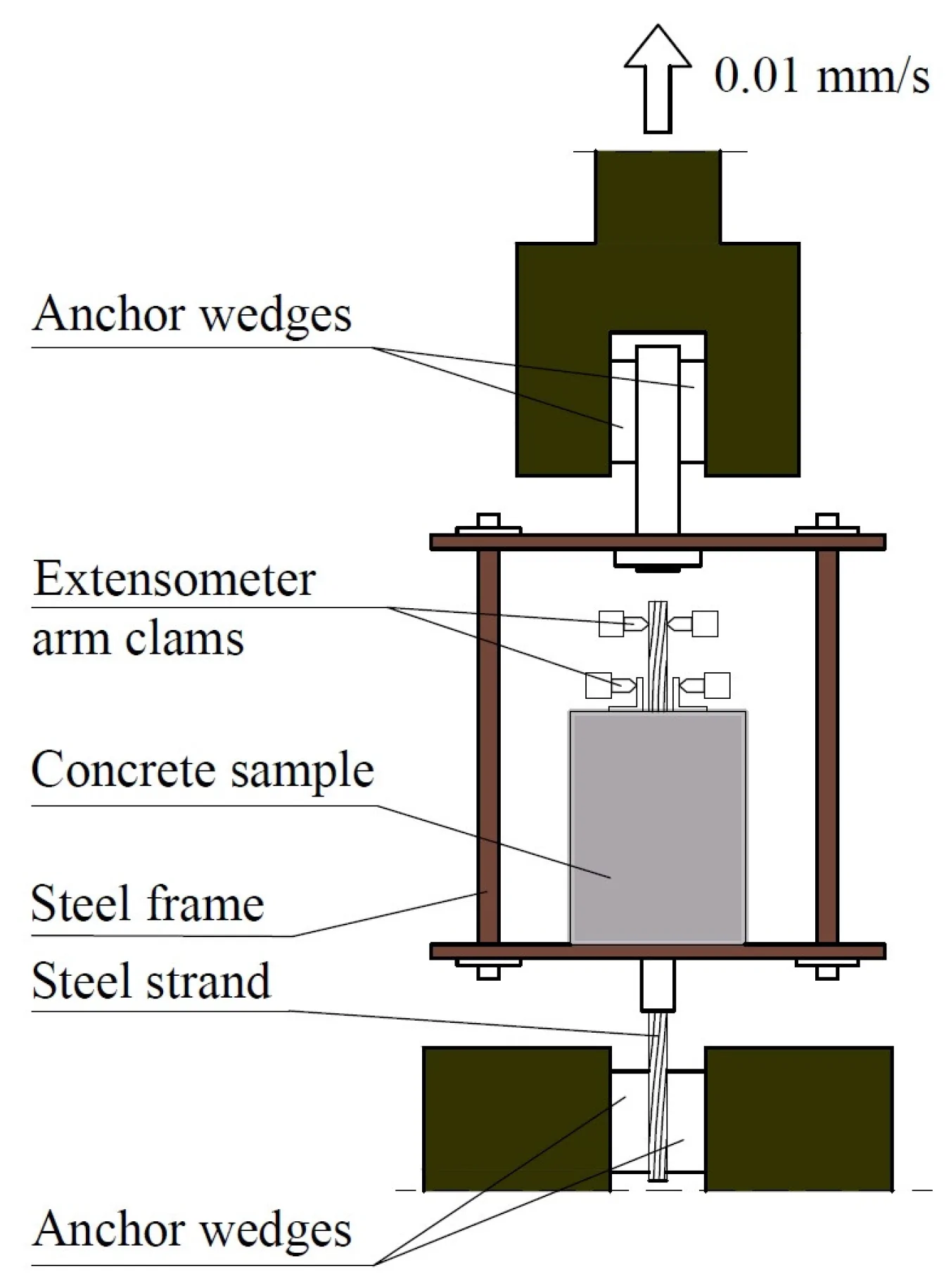
Scheme of testing machine [17].

**Figure 3:**
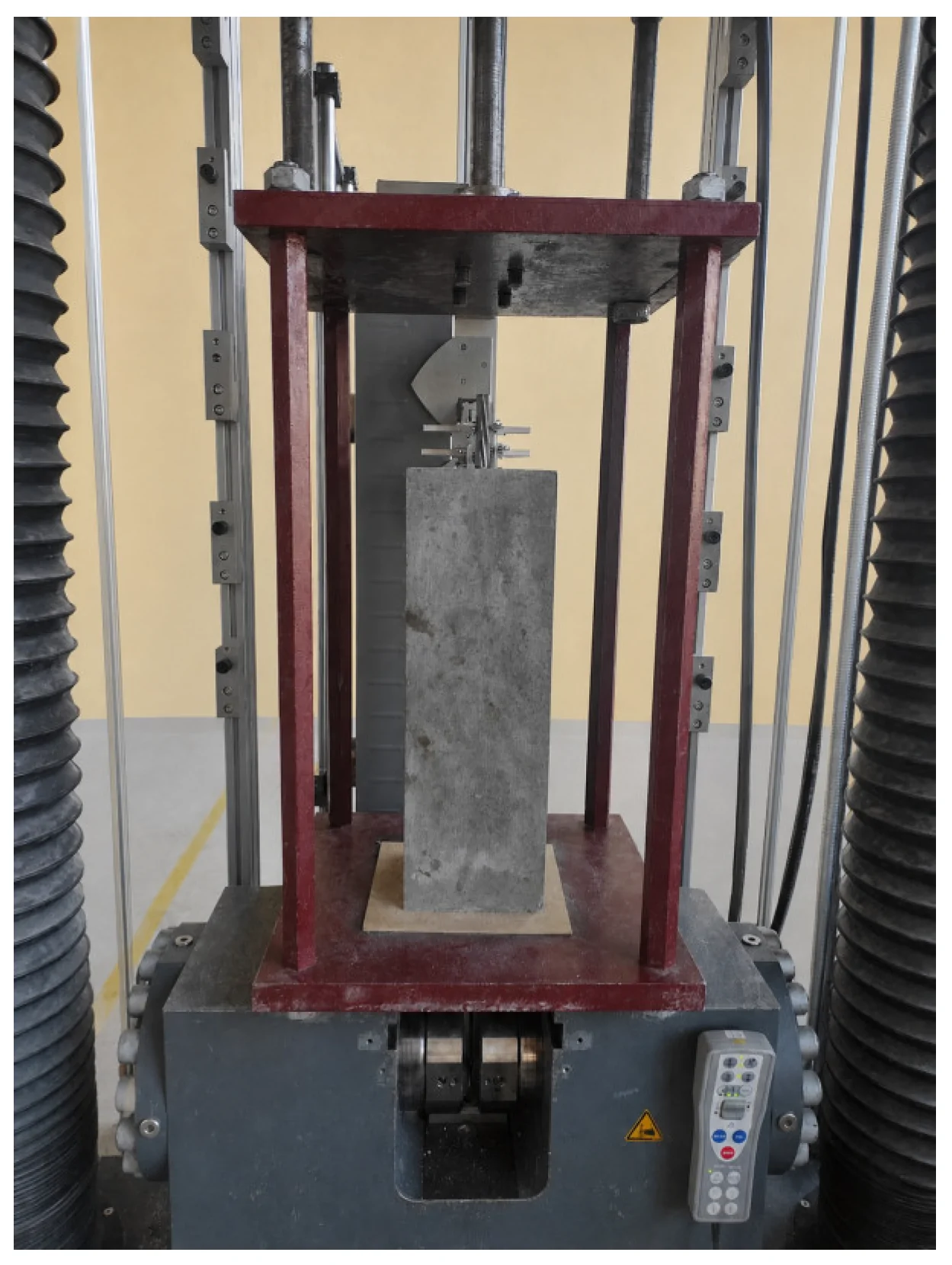
Extensometer mounted at the free end of a plain steel strand of 15.7 mm during testing.

The standards do not provide specific recommended methods for evaluating bond stress. The only standardized method available is outlined in [23], which relates to the testing of non-pretensioned strands for applicability. This method enables the determination of bond stress exclusively along the anchorage length of the non-pretensioned steel strand. In the existing literature, numerous alternative methods have been developed by various researchers. This study introduces a modified pull-out testing method devised by the author. This method permits the determination of the actual bond stress value (including adhesive bond) at the moment of prestressing force release. Additionally, this method is applicable to both pretensioned and non-pretensioned steel strands. The specimens used to investigate the bond stress–slip relationship with axially embedded steel prestressing strands were placed within a specially constructed steel frame (Figure 2). This frame consists of two rigid steel plates, referred to as the upper and lower plates, connected by four steel hangers. The lower steel plate is equipped with a centrally drilled hole that permits the strand from the concrete specimen to pass through. A longer segment of the lower strand is secured in the gripping jaws of an advanced testing machine (Zwick-Roell Z1200, ZwickRoell GmbH & Co. KG, Ulm, Germany). The force generated by the steering mechanism raises the frame structure, thereby applying the load onto the concrete specimen through pressure on the bottom surface. The loading rate of 0.01 mm/s controlled the pushing load via displacement, while the recording system continuously captured the force value in a digital format. The relative displacement corresponding to the specified force was measured using a system consisting of two arms of the incremental extensometer (Figure 3). The upper arms recorded displacement at the free end of the steel strand, while the lower arms measured the displacement of the aluminum angles bonded to the concrete surface in relation to their original position. The final value displayed on the computer screen indicates the relative displacement between the two materials. The margin of error for the extensometer readings is 0.12 μm. The research continued until the displacement value predetermined in the program (based on preliminary tests) was achieved.

## 4. Results

The comprehensive results of the experimental studies conducted on specimens made from LWAC type A concrete in the Laboratory of the Institute of Materials and Building Structures are detailed in the work [17]. The compressive strength, splitting tensile strength, and modulus of elasticity of the concrete were assessed, with the findings displayed in Table 6.

The bond stress (f_b_) of lightweight aggregate concrete (LWAC) to non-pretensioned steel strands was calculated using Equation (1):

(1) f_b_ = F/(C · l_emb_)

where the circumference C of the strand was obtained from Relation (2):

(2) C = 4/3 · π · ϕ

The relationship between force values and slip recorded by the electronic system for each test specimen allowed for the identification of the force value at which concrete lost its adherence to the prestressing strand. Furthermore, pushing force values were documented at the following slip measurements: 0.01, 0.0254, 0.10, 0.254, 1.0, 2.0, 2.54, 3, 4, 5, 6, 7, 8, 9, 10, 11, 12, 13, 14, and 15 mm. It is noteworthy that for LWAC type A specimens, slip was only measured up to 10 mm. The average pushing force values associated with the specified slip measurements for each embedment length of a 15.7 mm diameter steel strand, which correspond to the concrete compressive strengths after 3 and 28 days of curing, are presented in Table 4 and Table 6 for LWAC type A specimens, and in Table 7 and Table 8 for LWAC type B specimens [17].

The summarized average bond stress values for LWAC type A with non-pretensioned steel strands, associated with concrete compressive strengths of 35.6 MPa and 55.3 MPa, can be found in Table 7 and Table 9, respectively [17]. Furthermore, the average bond stress values for LWAC type B in relation to non-pretensioned steel strands at compressive strengths of 42.4 MPa and 57.7 MPa are recorded in Table 9 and Table 10, respectively. These tables also provide the maximum bond stress values, along with average bond stress values for slip ranges of 1 to 10 mm and 1 to 15 mm (in the case of LWAC type B specimens), as well as the coefficients of variation (CoV).

## 5. Discussion of Test Findings

**Figure 4:**
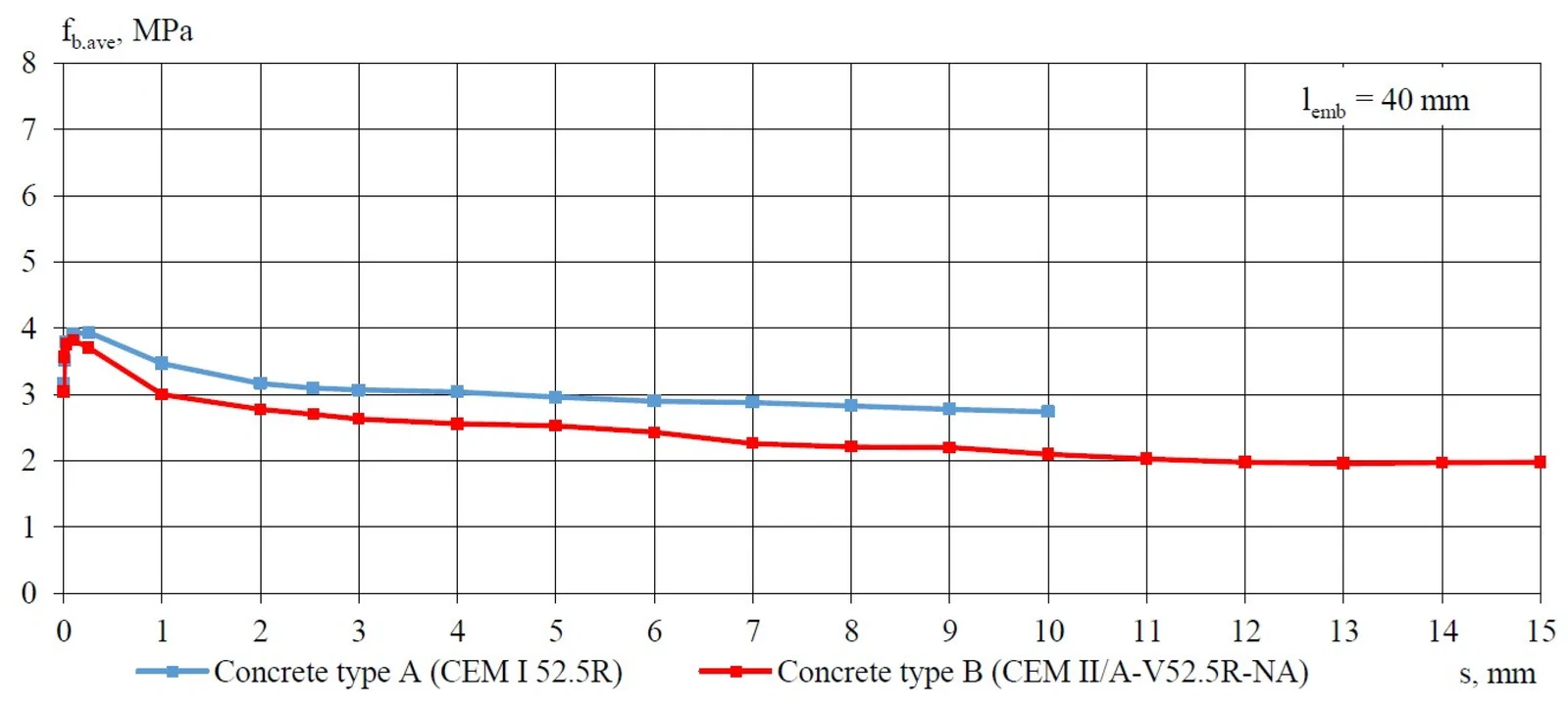
Average bond stress–slip relationship (*f_b,ave_ − s*) for LWAC specimen with cross-section 160 × 160 mm and non-pretensioned plain seven-wire steel strand of 15.7 mm diameter and l_emb_ = 40 mm for 2 types of concrete (A and B) on day 3 after curing.

**Figure 5:**
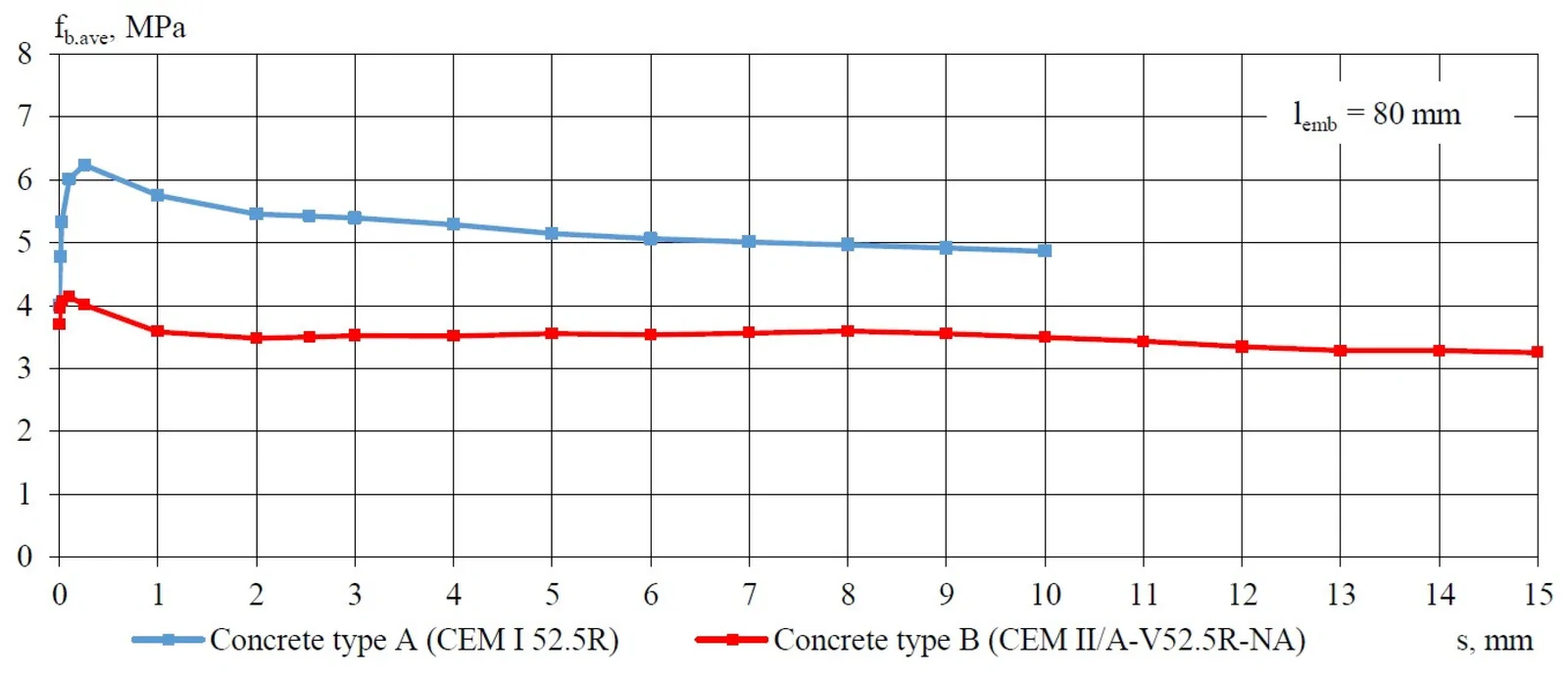
Average bond stress–slip relationship (*f_b,ave_* − *s*) for LWAC specimen with cross-section 160 × 160 mm and non-pretensioned plain seven-wire steel strand of 15.7 mm diameter and l_emb_ = 80 mm for 2 types of concrete (A and B) on day 3 after curing.

**Figure 6:**
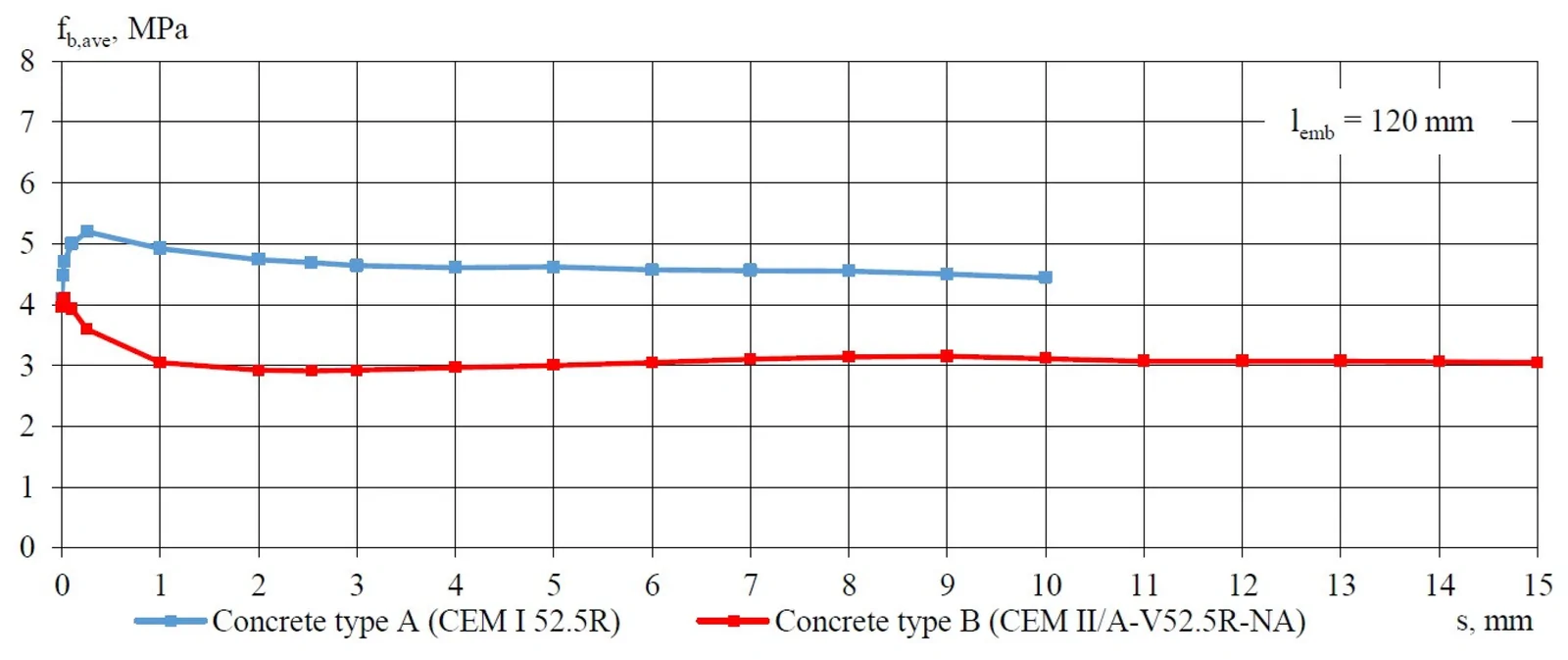
Average bond stress–slip relationship (*f_b,ave_* − *s*) for LWAC specimen with cross-section 160 × 160 mm and non-pretensioned plain seven-wire steel strand of 15.7 mm diameter and l_emb_ = 120 mm for 2 types of concrete (A and B) on day 3 after curing.

**Figure 7:**
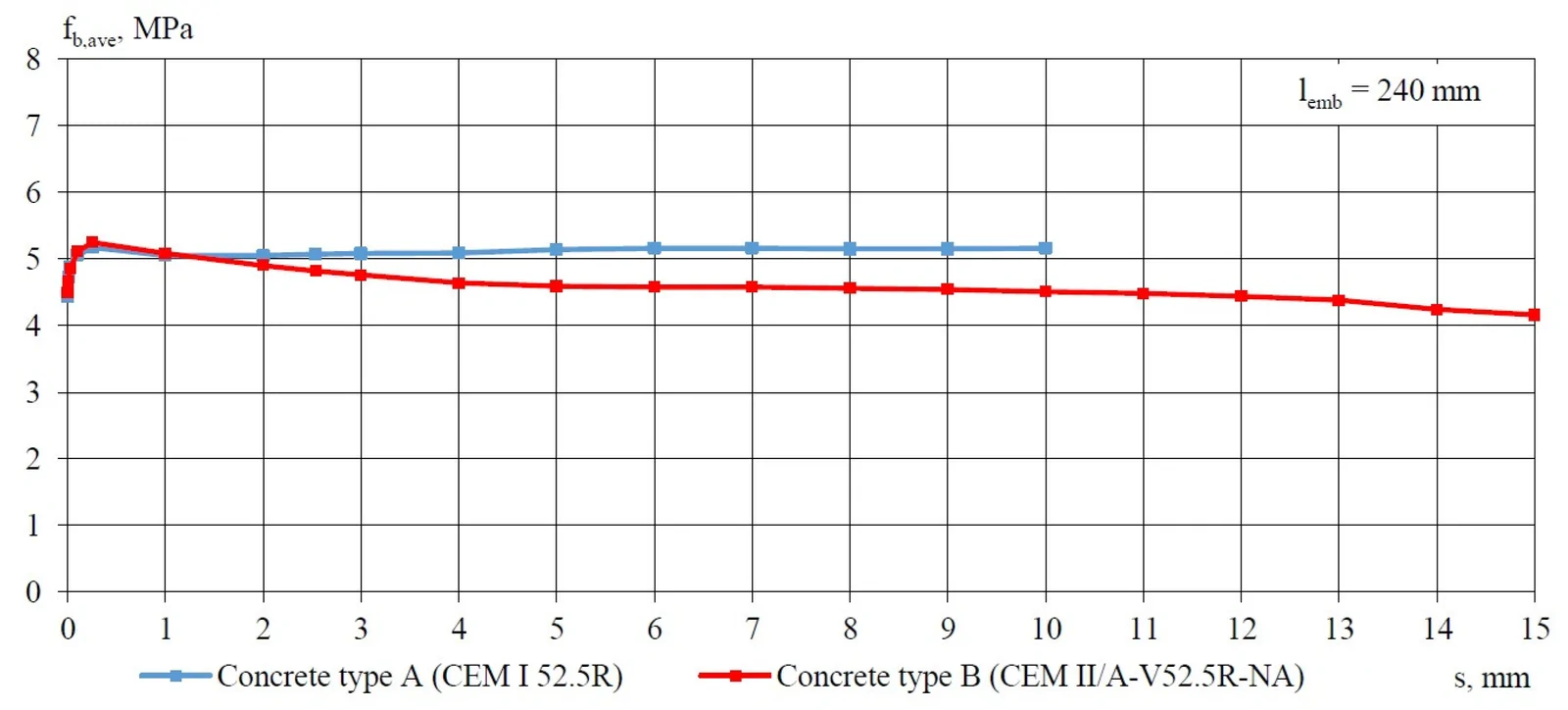
Average bond stress–slip relationship (*f_b,ave_* − *s*) for LWAC specimen with cross-section of 160 × 160 mm and non-pretensioned plain seven-wire steel strand of 15.7 mm diameter and l_emb_ = 240 mm for 2 types of concrete (A and B) on day 3 after curing.

**Figure 8:**
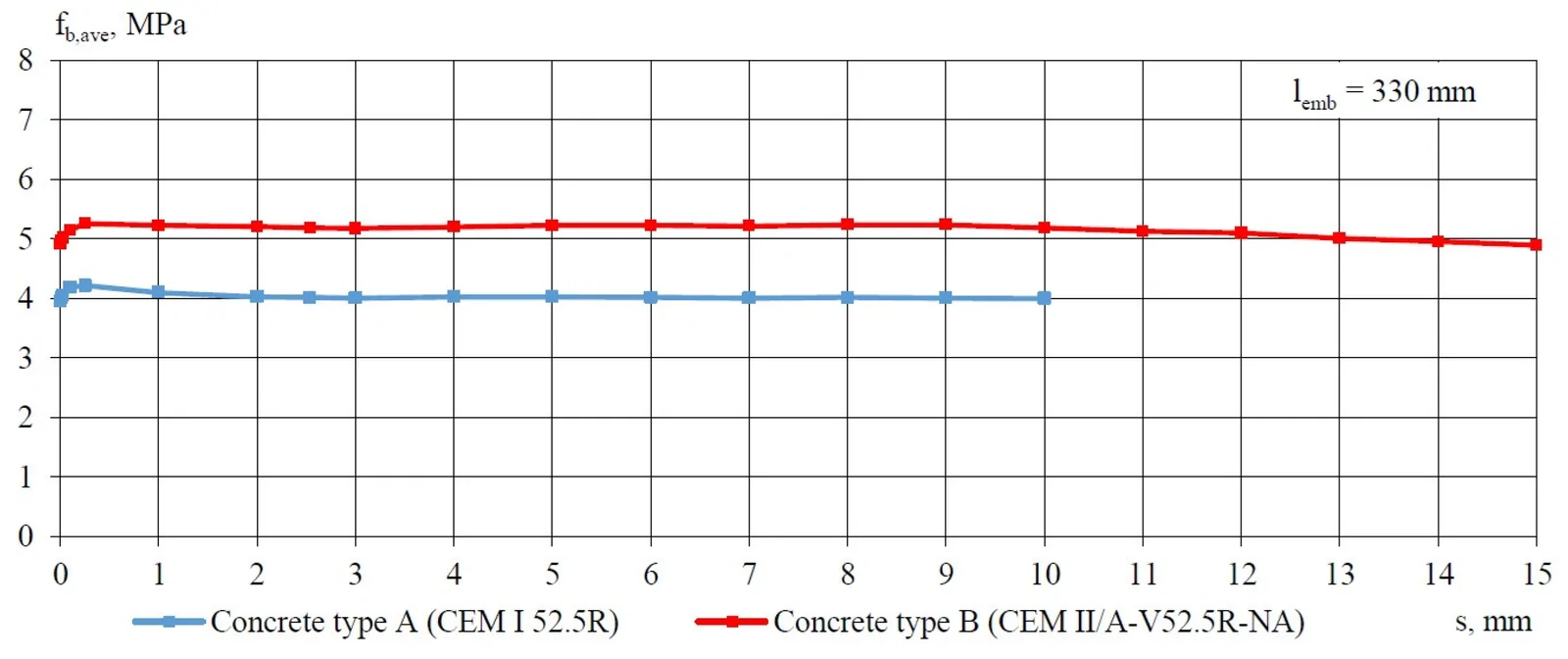
Average bond stress–slip relationship (*f_b,ave_* − *s*) for LWAC specimen with cross-section 160 × 160 mm and non-pretensioned plain seven-wire steel strand of 15.7 mm diameter and l_emb_ = 330 mm for 2 types of concrete (A and B) on day 3 after curing.

**Figure 9:**
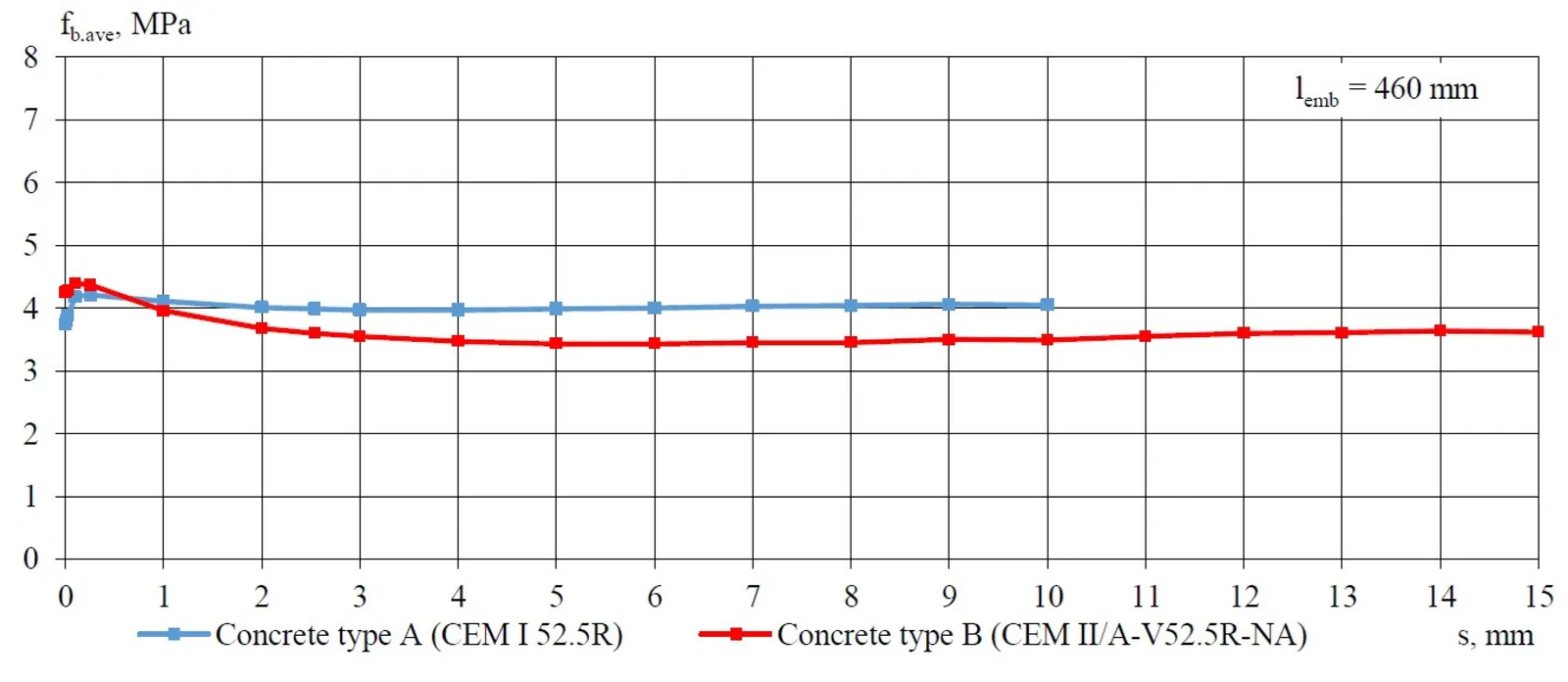
Average bond stress–slip relationship (*f_b,ave_* − *s*) for LWAC specimen with cross-section 160 × 160 mm and non-pretensioned plain seven-wire steel strand of 15.7 mm diameter and l_emb_ = 460 mm for 2 types of concrete (A and B) on day 3 after curing.

**Figure 10:**
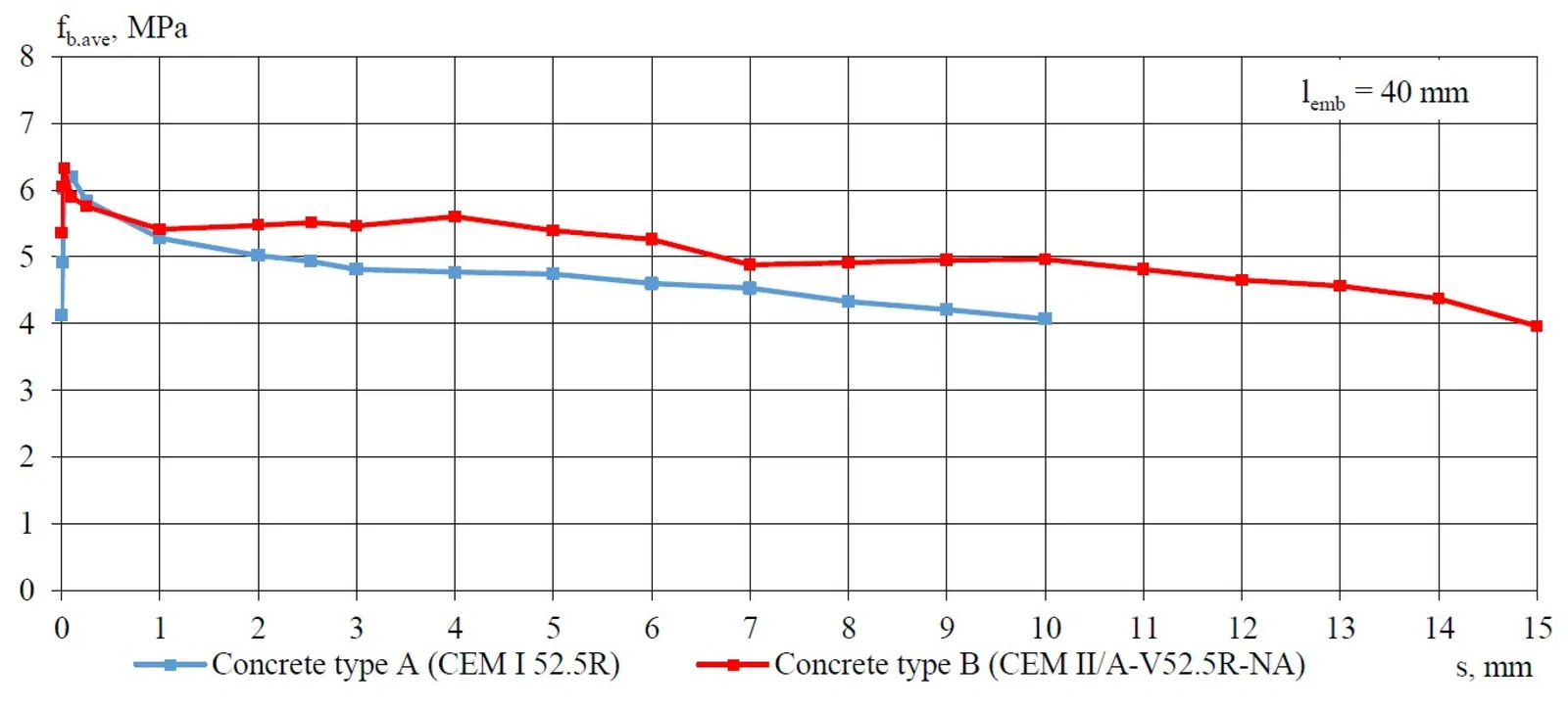
Average bond stress–slip relationship (*f_b,ave_* − *s*) for LWAC specimen with cross-section 160 × 160 mm and non-pretensioned plain seven-wire steel strand of 15.7 mm diameter and l_emb_ = 40 mm for 2 types of concrete (A and B) on day 28 after curing.

**Figure 11:**
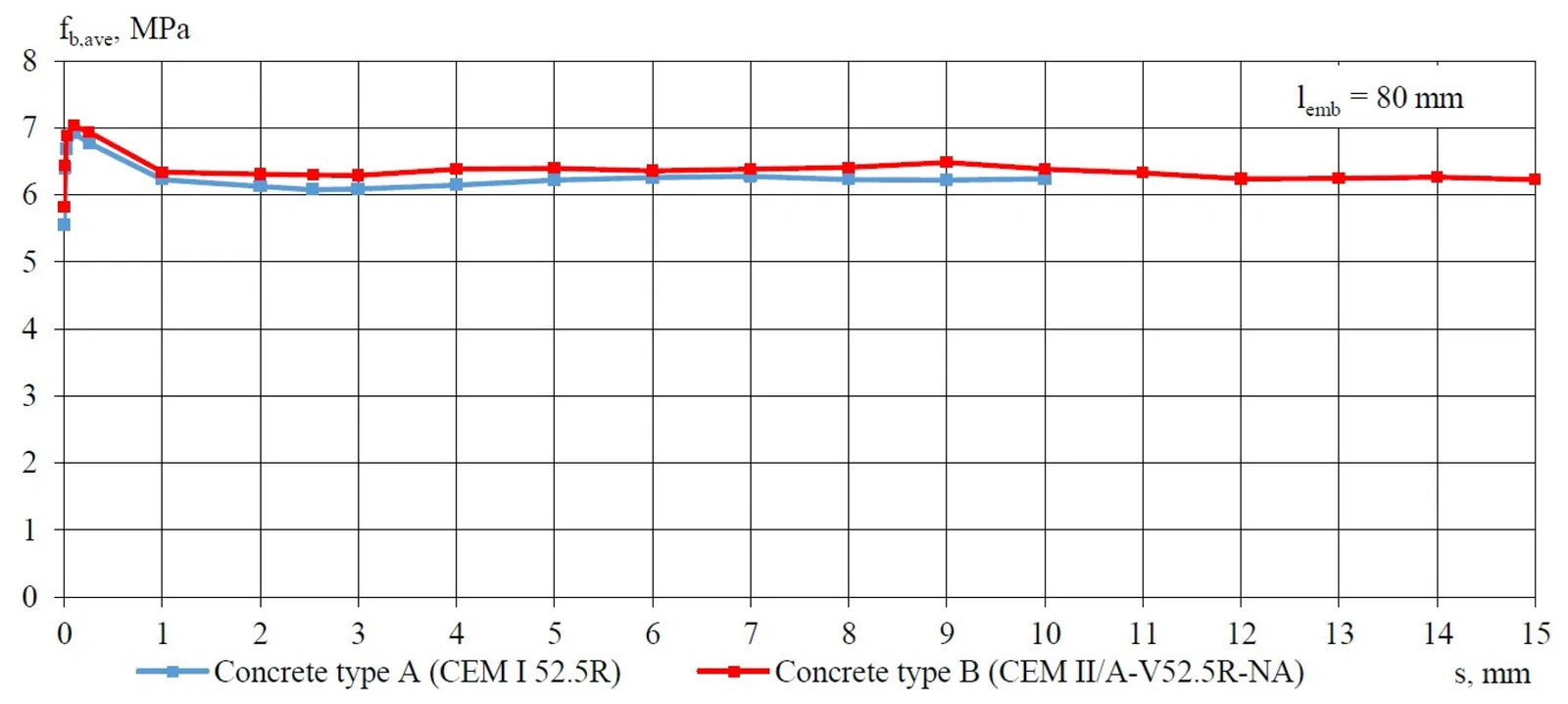
Average bond stress–slip relationship (*f_b,ave_* − *s*) for LWAC specimen with cross-section 160 × 160 mm and non-pretensioned plain seven-wire steel strand of 15.7 mm diameter and l_emb_ = 80 mm for 2 types of concrete (A and B) on day 28 after curing.

**Figure 12:**
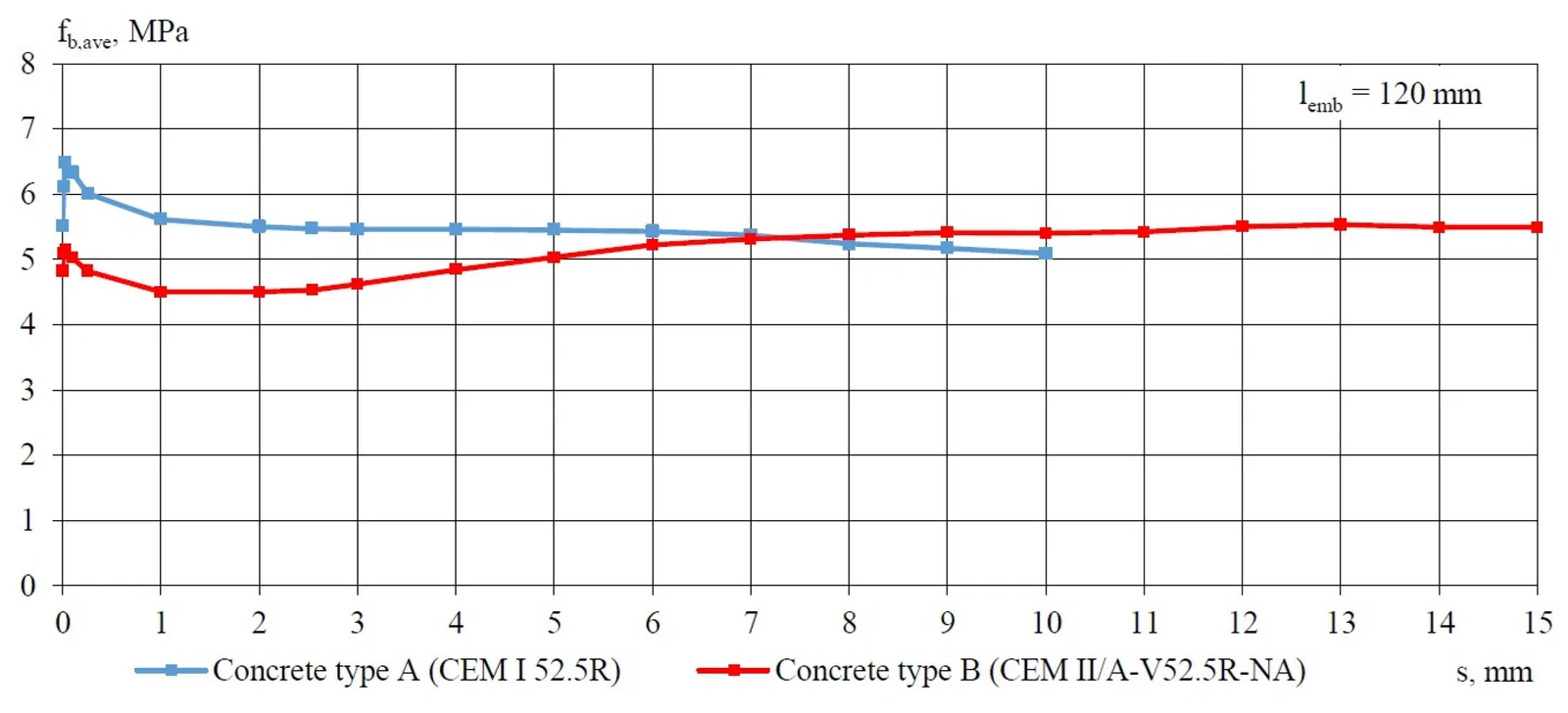
Average bond stress–slip relationship (*f_b,ave_* − *s*) for LWAC specimen with cross-section 160 × 160 mm and non-pretensioned plain seven-wire steel strand of 15.7 mm diameter and l_emb_ = 120 mm for 2 types of concrete (A and B) on day 28 after curing.

**Figure 13:**
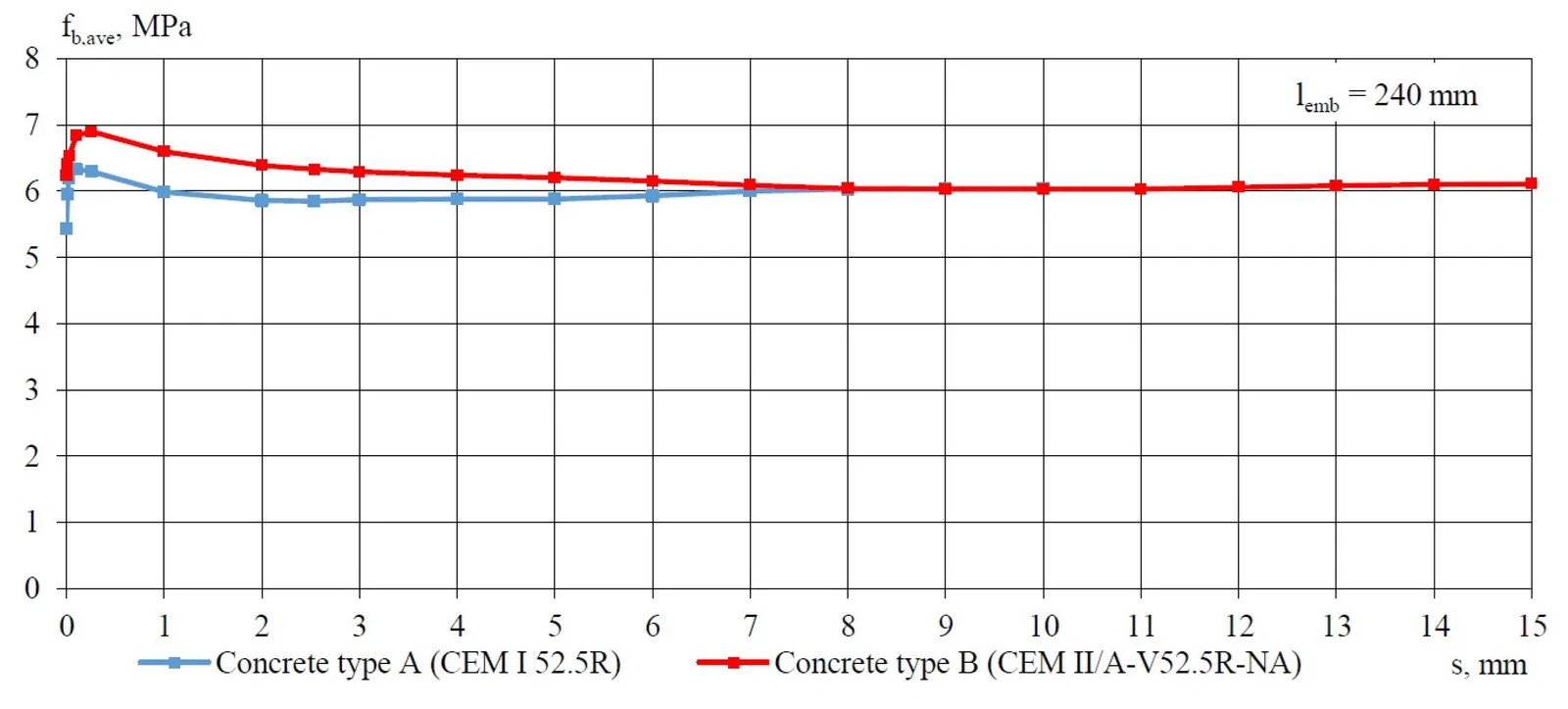
Average bond stress–slip relationship (*f_b,ave_* − *s*) for LWAC specimen with cross-section 160 × 160 mm and non-pretensioned plain seven-wire steel strand of 15.7 mm diameter and l_emb_ = 240 mm for 2 types of concrete (A and B) on day 28 after curing.

**Figure 14:**
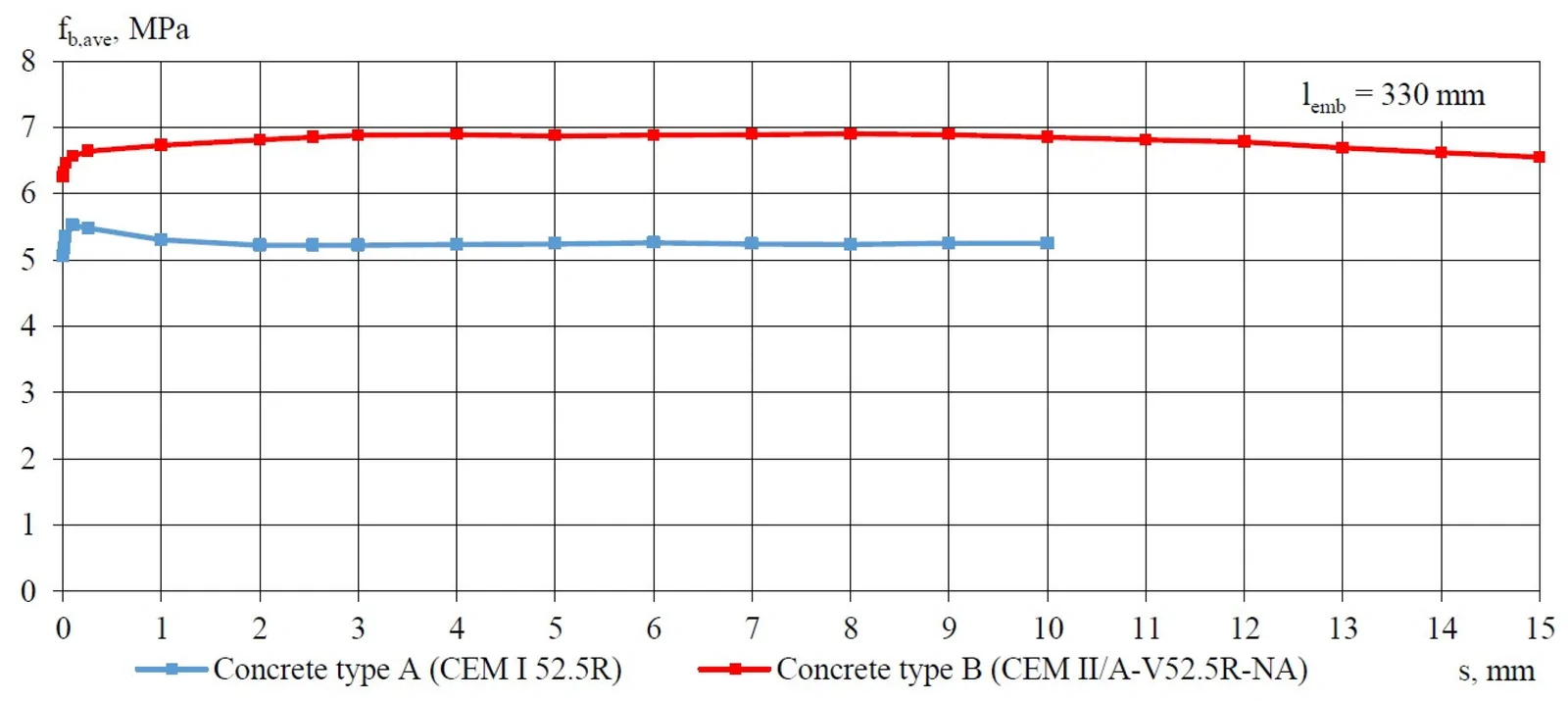
Average bond stress–slip relationship (*f_b,ave_* − *s*) for LWAC specimen with cross-section 160 × 160 mm and non-pretensioned plain seven-wire steel strand of 15.7 mm diameter and l_emb_ = 330 mm for 2 types of concrete (A and B) on day 28 after curing.

**Figure 15:**
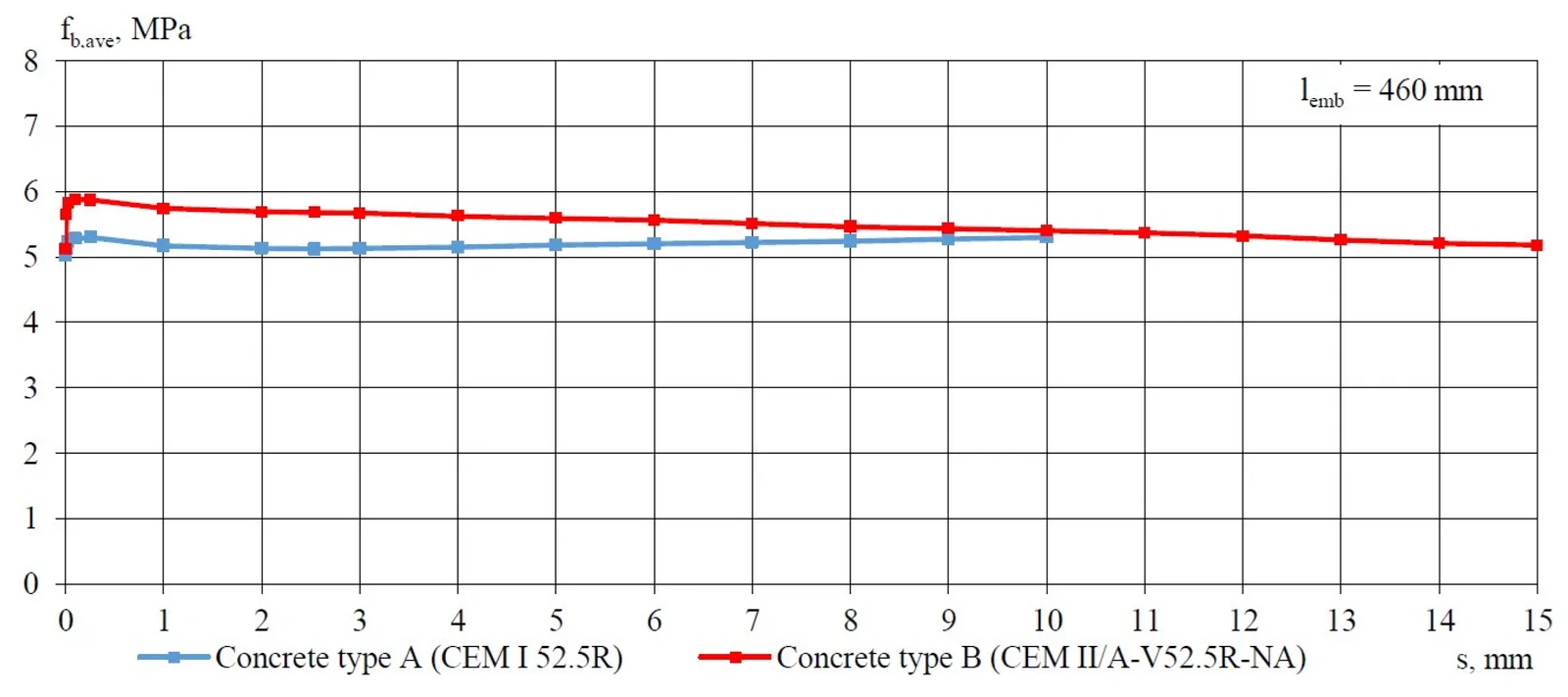
Average bond stress–slip relationship (*f_b,ave_* − *s*) for LWAC specimen with cross-section 160 × 160 mm and non-pretensioned plain seven-wire steel strand of 15.7 mm diameter and l_emb_ = 460 mm for 2 types of concrete (A and B) on day 28 after curing.

In order to assess the effectiveness of both types of cement for the manufacture of precast prestressed concrete elements, a comparative analysis was performed on the distributions of average bond stress in relation to the slip of non-pretensioned steel strand with a diameter of 15.7 mm. Figure 4, Figure 5, Figure 6, Figure 7, Figure 8 and Figure 9 illustrate the analyzed distributions of f_b,ave-s_ determined for bond lengths of 40, 80, 120, 240, 330, and 460 mm after 3 days of concrete curing. Corresponding distributions of f_b,ave-s_ for bond lengths of 40, 80, 120, 240, 330, and 460 mm after 28 days of curing are presented in Figure 10, Figure 11, Figure 12, Figure 13, Figure 14 and Figure 15.

The analysis of the distributions of f_b,ave-s_ indicates that for bond lengths of 40, 80, and 120 mm, a significant increase in bond stress is observed following the loss of adhesive bond, reaching a maximum at a slip of the strand that is less than or equal to 0.254 mm. Once the slip exceeds 1 mm, a certain stabilization of the average bond stress is noted for both types A and B of concrete. This phenomenon is evident in test specimens assessed after both 3 and 28 days of concrete curing. In the case of test specimens with a bond length of 40 mm, a gradual reduction in bond stress is observed after achieving the maximum average bond stress as the slip value of the strand increases.

In the case of bond lengths measuring 80 mm and 120 mm, the residual bond stresses calculated for slip within the range of 1 to 10 mm (Table 11 and Table 12) on test specimens made of type A concrete are, respectively, 47% and 52% higher than the corresponding values obtained from test specimens made of type B concrete after 3 days of concrete curing. For specimens assessed after 28 days of curing, the average bond stress value difference does not exceed 8%.

Regarding specimens with bond lengths of 240 mm, 330 mm, and 460 mm, once the adhesive bond is surpassed, there is an increase in the average bond stress value until it reaches its peak, followed by a reduction in the average bond stress value, while simultaneously stabilizing within the slip range of 1 to 10 mm or 1 to 15 mm. The reduction in the average bond stress value within the slip range of 1 to 10 mm, as observed in specimens made of type A concrete, does not exceed 10% after both 3 and 28 days of curing. Conversely, for specimens made of type B concrete, the reduction in the average bond stress value in the slip range of 1 to 10 mm is limited to 24% and 12% after 3 and 28 days of curing, respectively.

**Figure 16:**
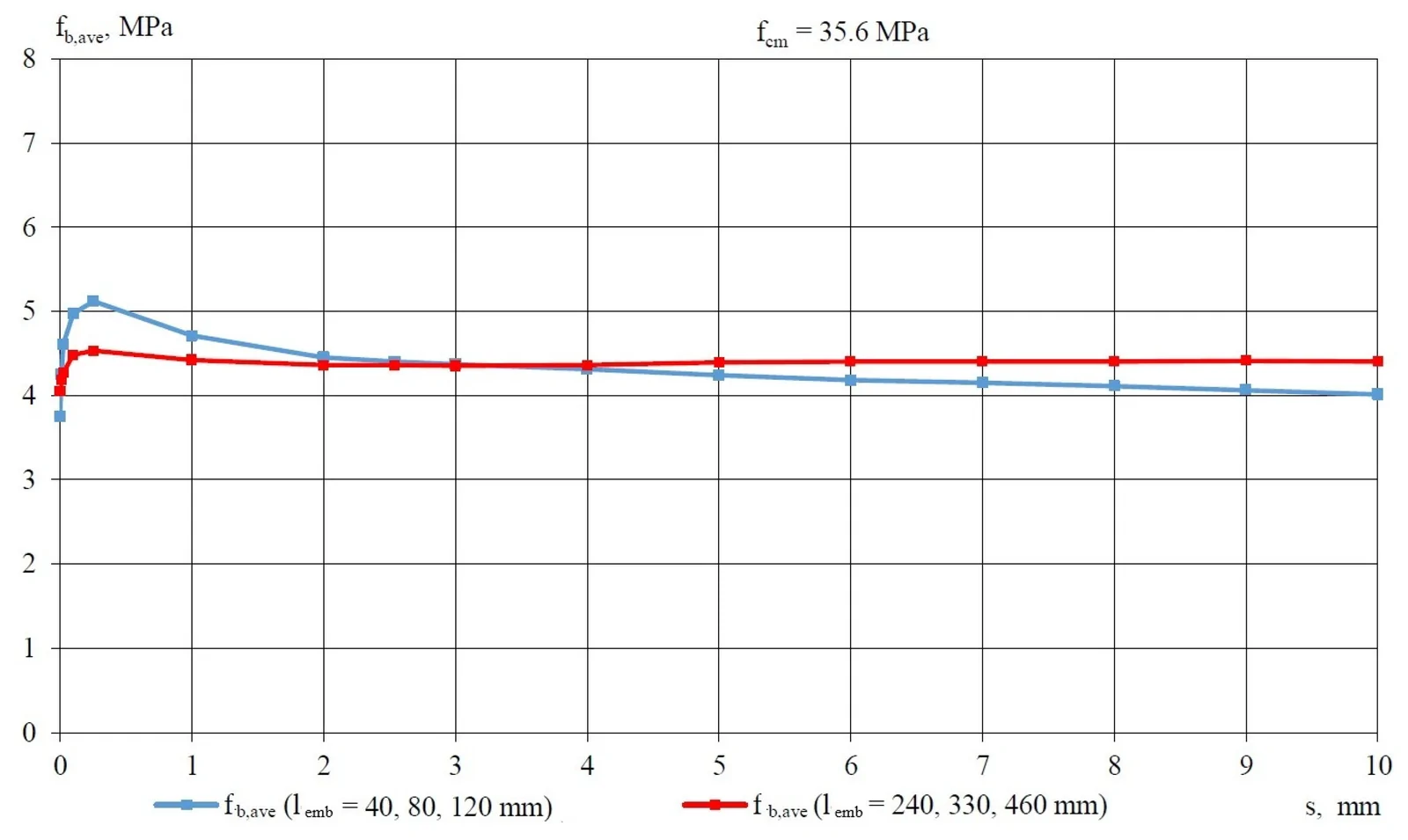
Average bond stress–slip relationship (*f_b,ave_* − *s*) for LWAC specimen with cross-section 160 × 160 mm and non-pretensioned plain seven-wire steel strand of 15.7 mm diameter for two cases of l_emb_, f_cm_ = 35.6 MPa.

**Figure 17:**
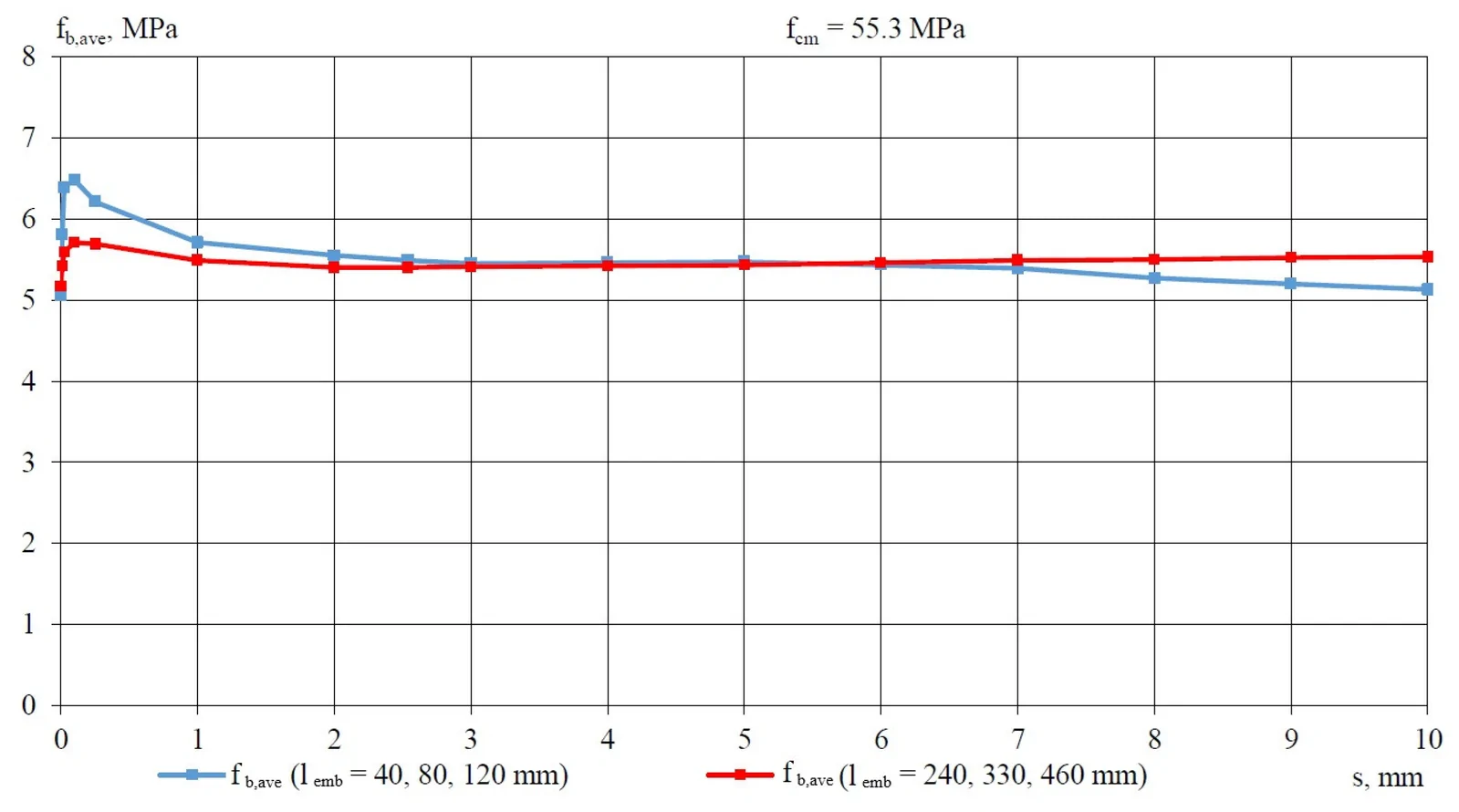
Average bond stress–slip relationship (*f_b,ave_* − *s*) for LWAC specimen with cross-section 160 × 160 mm and non-pretensioned plain seven-wire steel strand of 15.7 mm diameter for two cases of l_emb_, f_cm_ = 55.3 MPa.

**Figure 18:**
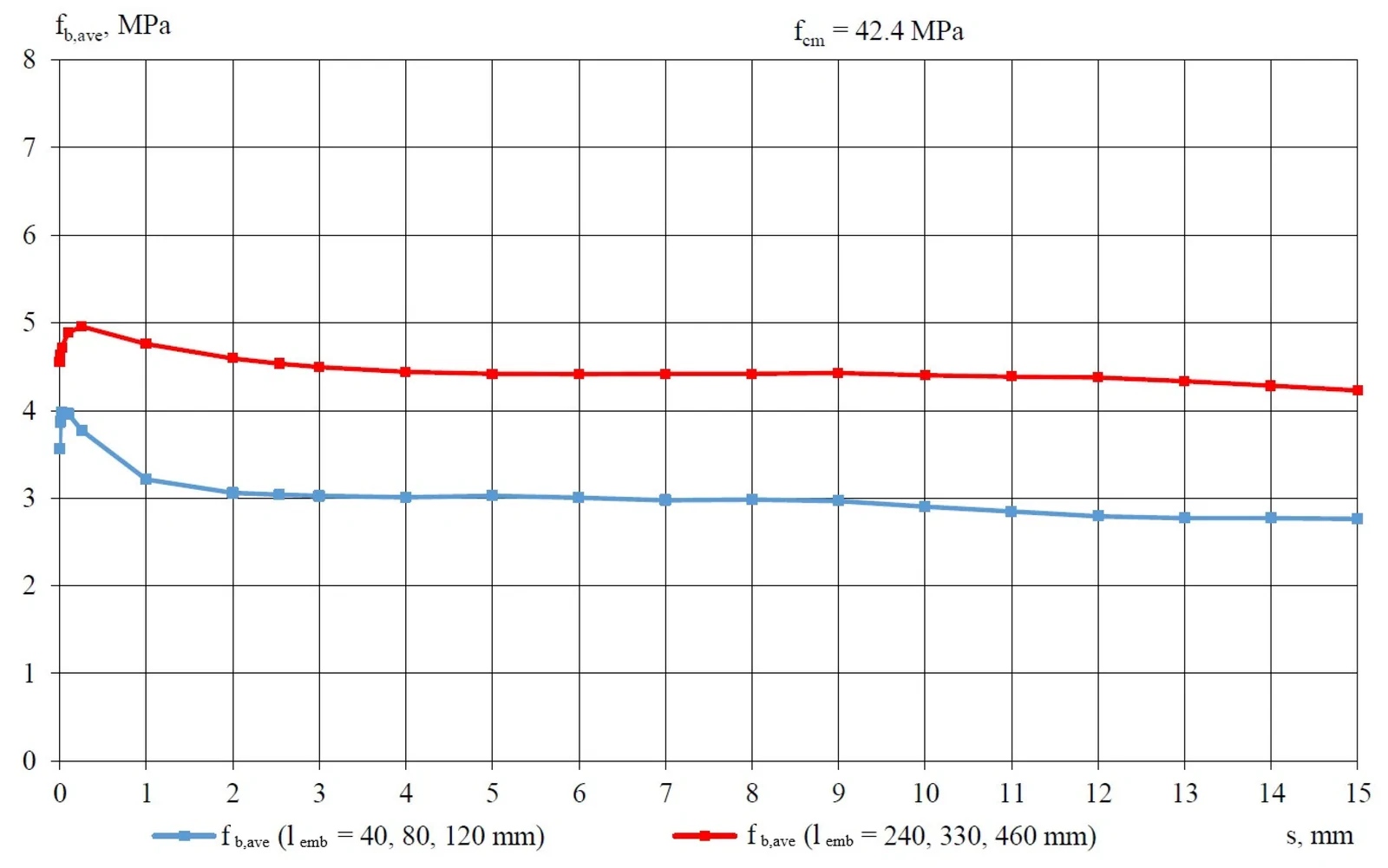
Average bond stress–slip relationship (*f_b,ave_* − *s*) for LWAC specimen with cross-section 160 × 160 mm and non-pretensioned plain seven-wire steel strand of 15.7 mm diameter for two cases of l_emb_, f_cm_ = 42.4 MPa.

**Figure 19:**
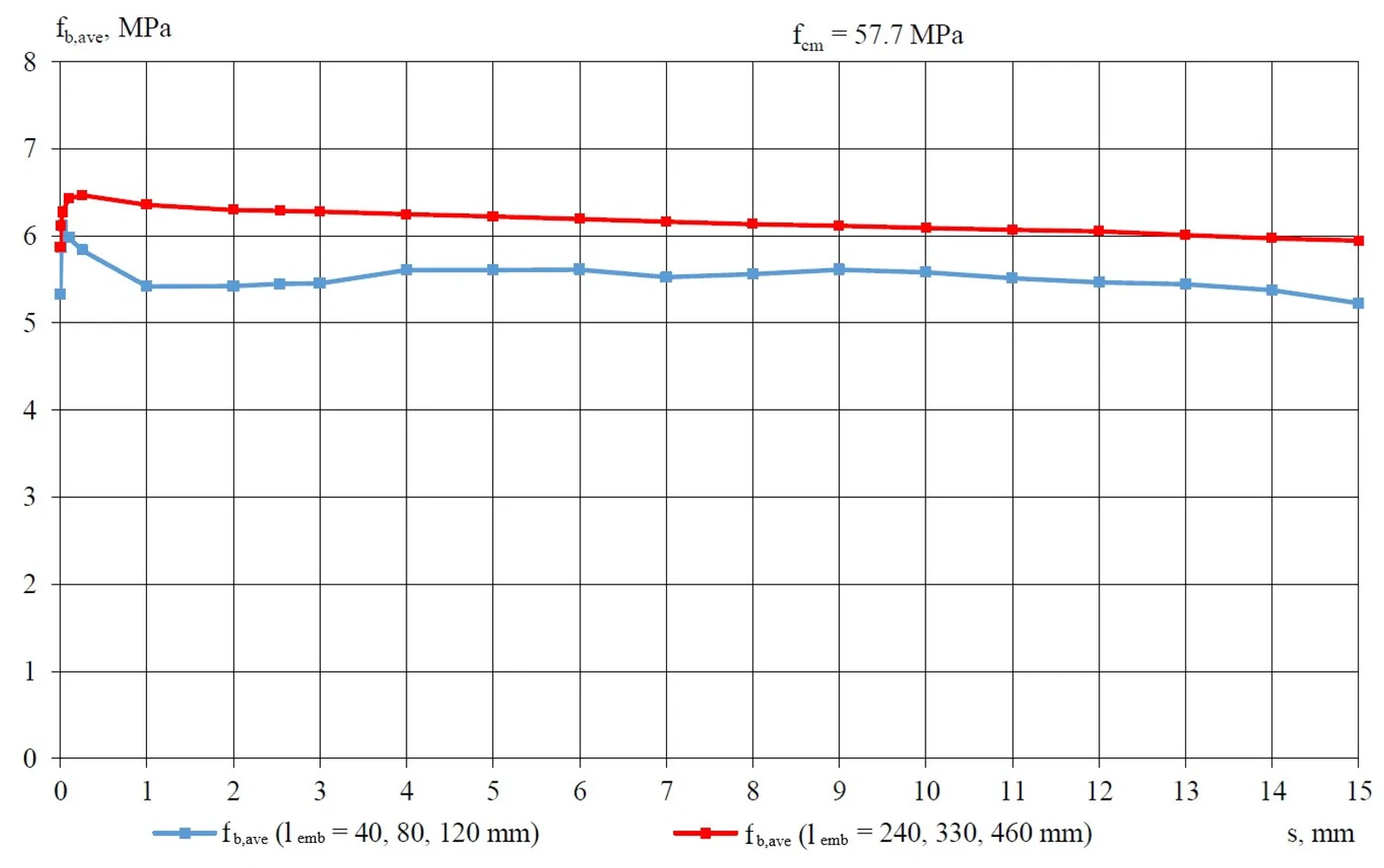
Average bond stress–slip relationship (*f_b,ave_* − *s*) for LWAC specimen with cross-section 160 × 160 mm and non-pretensioned plain seven-wire steel strand of 15.7 mm diameter for two cases of l_emb_, f_cm_ = 57.7 MPa.

The following discussion involves an analysis of the results obtained, which were divided into two categories based on bond lengths: 40 to 120 mm and 240 to 460 mm. The average bond stress values calculated after 3 and 28 days of concrete maturation for specimens made from type A and type B concrete are summarized in Table 11 and Table 12, respectively. Furthermore, these tables present the maximum bond stress values, the average bond stress values for slips 1 ÷ 10 and 1 ÷ 15, as well as the bond stress values where adhesion loss was observed. Although type B concrete exhibited higher strength, the bond stresses between the concrete and a non-pretensioned steel strand with a bond length (l_emb_) of 40 to 120 mm were considerably lower than the corresponding f_b_ values for type A concrete. The distributions of the average bond stress–slip relationship for lightweight aggregate concrete type A specimens, with a cross-section of 160 × 160 mm and a non-pretensioned plain seven-wire steel strand of ϕ15.7 mm diameter, for two different embedment lengths are illustrated in Figure 16 (for f_cm_ = 35.6 MPa) and Figure 17 (for f_cm_ = 55.3 MPa). Similar distributions of f_b,ave-s_ relationships were obtained for type B concrete, as shown in Figure 18 (for f_cm_ = 42.4 MPa) and Figure 19 (for f_cm_ = 57.7 MPa).

**Table 11:** Average values of bond stress for LWAC type A to plain seven-wire steel strand for specimens with two separated l_emb_ groups.

s mm	f_cm_ = 35.6 MPa		f_cm_ = 55.3 MPa	
	l_emb_ = 40, 80, 120 mm	l_emb_ = 240, 330, 460 mm	l_emb_ = 40, 80, 120 mm	l_emb_ = 240, 330, 460 mm
	f_b.ave_ MPa	f_b.ave_ MPa	f_b.ave_ MPa	f_b.ave_ MPa
adhesion	3.75	4.05	5.06	5.17
0.01	4.25	4.18	5.81	5.42
0.0254	4.61	4.27	6.39	5.59
0.1	4.97	4.48	6.48	5.71
0.254	5.12	4.53	6.21	5.69
1	4.71	4.42	5.71	5.49
2	4.45	4.36	5.55	5.40
2.54	4.40	4.36	5.49	5.40
3	4.37	4.35	5.45	5.41
4	4.31	4.36	5.46	5.42
5	4.24	4.39	5.47	5.43
6	4.18	4.40	5.43	5.46
7	4.15	4.40	5.39	5.49
8	4.11	4.40	5.27	5.50
9	4.06	4.41	5.20	5.52
10	4.01	4.40	5.13	5.53
*f_b max,ave_*	5.17	4.67	6.79	5.86
f_b.ave_ s = 1 ÷ 10 mm, MPa	4.27	4.39	5.41	5.46
f_b.ave_ s = 2 ÷ 10 mm, MPa	4.23	4.38	5.38	5.46

**Table 12:** Average values of bond stress for the LWAC type B to plain seven-wire steel strand for specimens with two separated l_emb_ groups.

s mm	f_cm_ = 42.4 MPa		f_cm_ = 57.2 MPa	
	l_emb_ = 40, 80, 120 mm	l_emb_ = 240, 330, 460 mm	l_emb_ = 40, 80, 120 mm	l_emb_ = 240, 330, 460 mm
	f_b.ave_ MPa	f_b.ave_ MPa	f_b.ave_ MPa	f_b.ave_ MPa
adhesion	3.56	4.55	5.33	5.87
0.01	3.87	4.63	5.86	6.12
0.0254	3.98	4.72	6.11	6.27
0.1	3.96	4.89	5.98	6.43
0.254	3.77	4.96	5.84	6.47
1	3.21	4.76	5.42	6.36
2	3.06	4.59	5.42	6.30
2.54	3.04	4.53	5.45	6.29
3	3.02	4.50	5.46	6.28
4	3.01	4.44	5.61	6.25
5	3.03	4.42	5.61	6.22
6	3.00	4.41	5.61	6.20
7	2.97	4.42	5.53	6.16
8	2.98	4.42	5.56	6.14
9	2.97	4.43	5.61	6.12
10	2.90	4.40	5.58	6.09
11	2.85	4.39	5.52	6.07
12	2.79	4.38	5.47	6.05
13	2.77	4.34	5.45	6.01
14	2.77	4.28	5.38	5.97
15	2.76	4.23	5.23	5.95
*f_b max,ave_*	4.16	5.02	6.42	6.63
f_b.ave_ s = 1 ÷ 10 mm, MPa	3.02	4.48	5.53	6.22
f_b.ave_ s = 1 ÷ 15 mm, MPa	2.95	4.43	5.49	6.15

**Figure 20:**
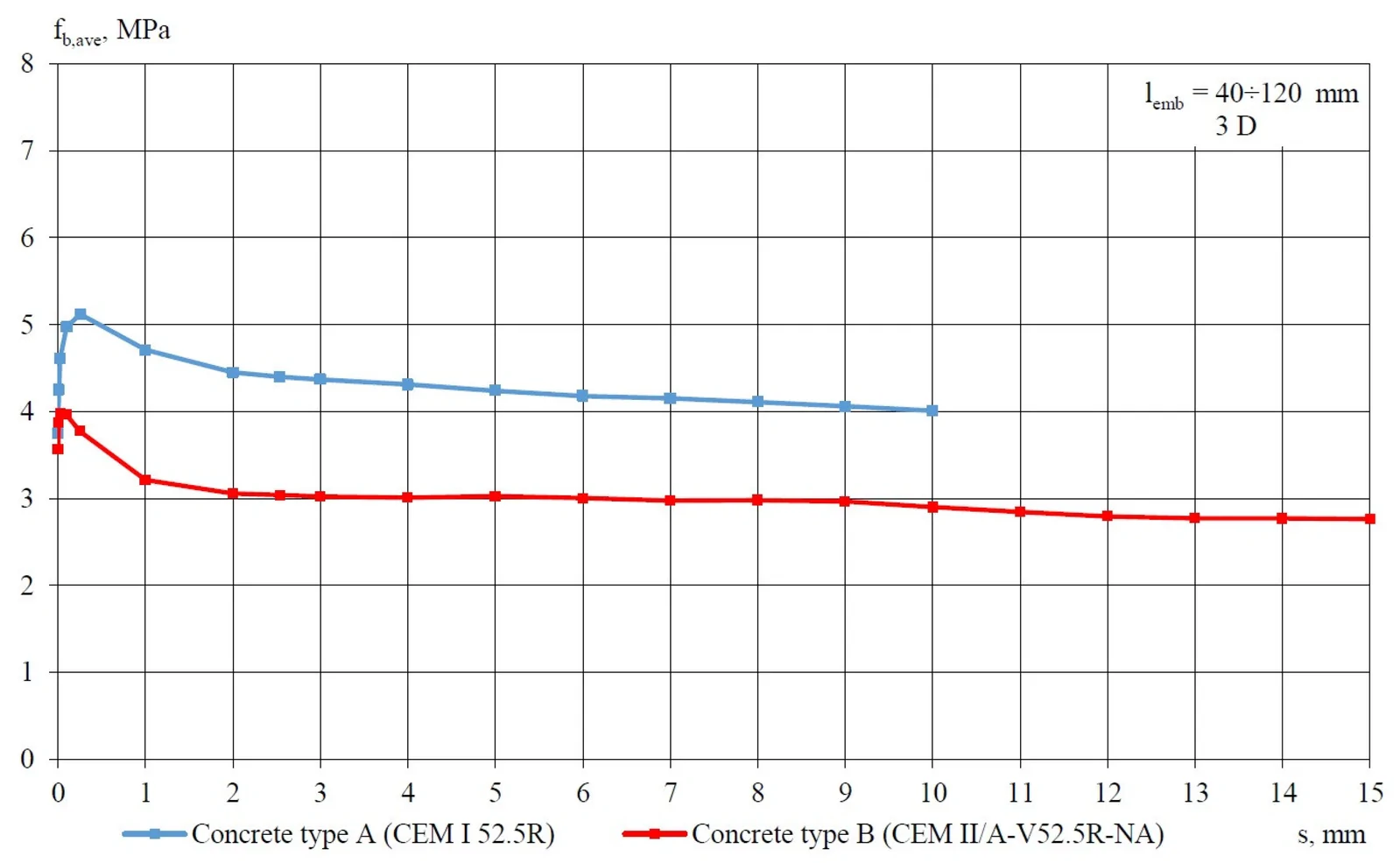
Average bond stress–slip (*f_b,ave_* − *s*) relationship for LWAC specimen with l_emb_ = 40 ÷ 120 mm and non-pretensioned plain seven-wire steel strand of 15.7 mm diameter, obtained after 3 days of curing for 2 types of concrete (A and B).

**Figure 21:**
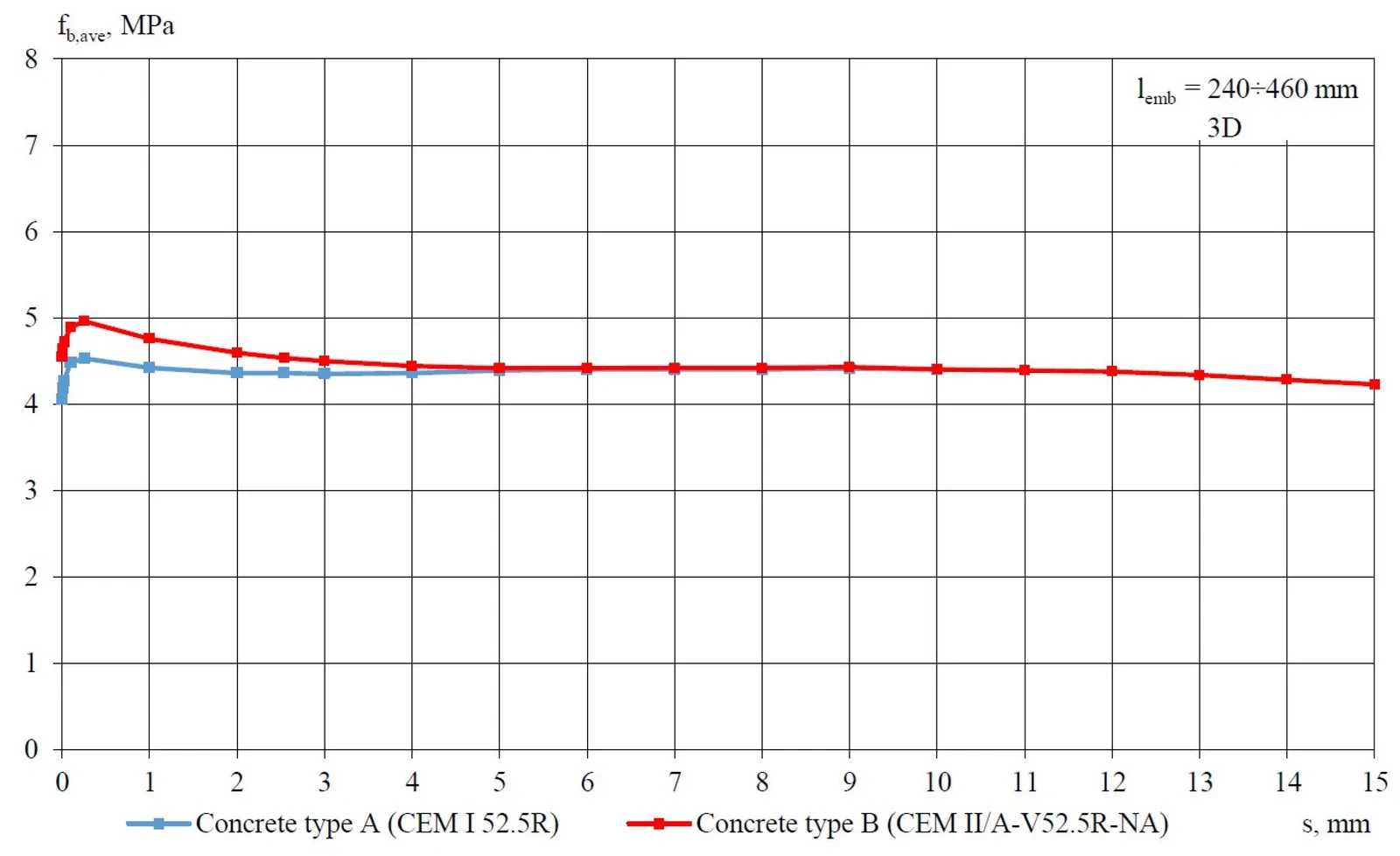
Average bond stress–slip (*f_b,ave_* − *s*) relationship for LWAC specimen with l_emb_ = 240 ÷ 460 mm and non-pretensioned plain seven-wire steel strand of 15.7 mm diameter, obtained after 3 days of curing for 2 types of concrete (A and B).

**Figure 22:**
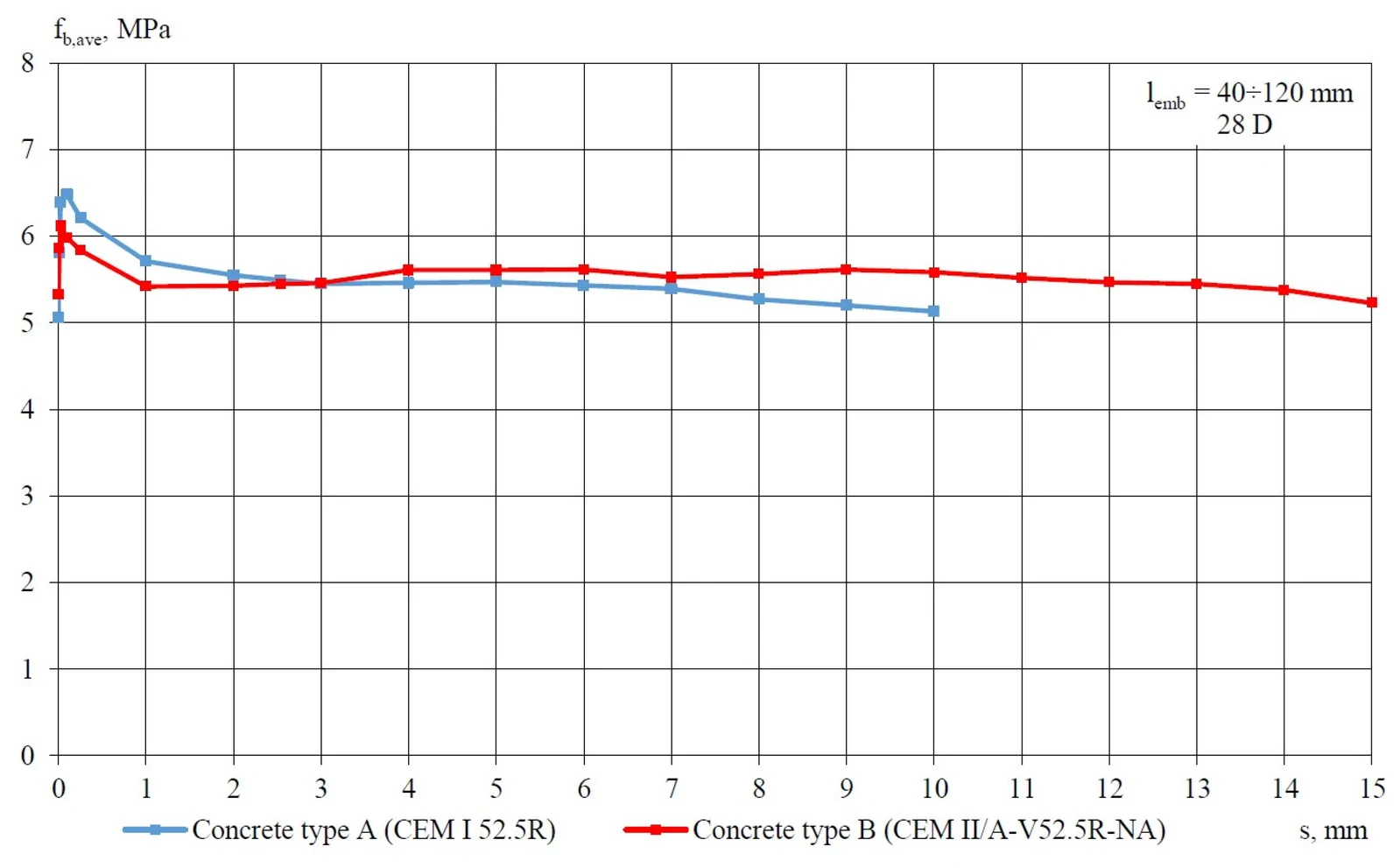
Average bond stress–slip (*f_b,ave_* − *s*) relationship for LWAC specimen with l_emb_ = 40 ÷ 120 mm and non-pretensioned plain seven-wire steel strand of 15.7 mm diameter, obtained after 28 days of curing for 2 types of concrete (A and B).

**Figure 23:**
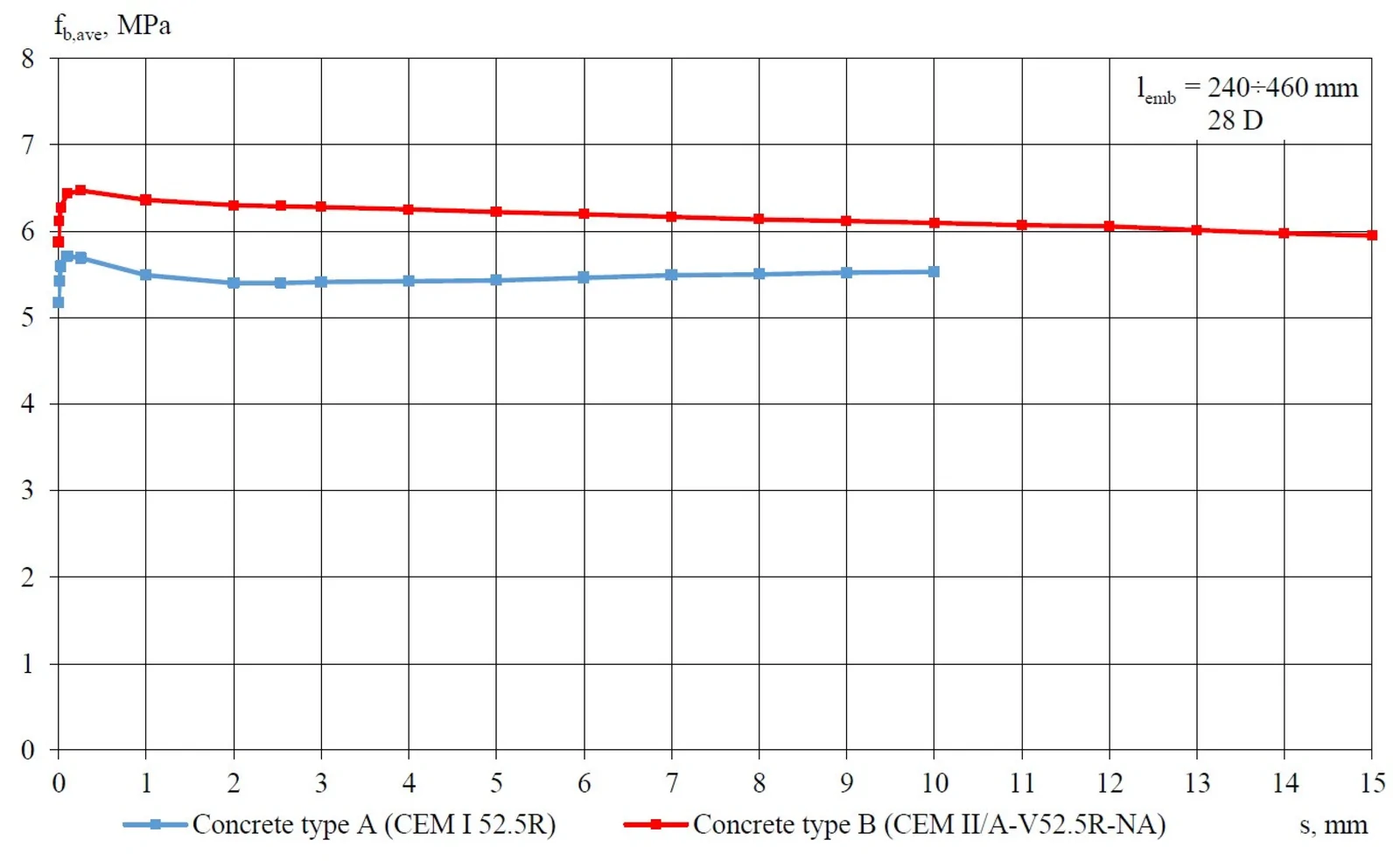
Average bond stress–slip (*f_b,ave_* − *s*) relationship for LWAC specimen with l_emb_ = 240 ÷ 460 mm and non-pretensioned plain seven-wire steel strand of 15.7 mm diameter, obtained after 28 days of curing for 2 types of concrete (A and B).

The distributions of the average bond stress–slip values obtained after 3 days of curing for both types A and B concrete, on test specimens with embedment lengths of l_emb_ = 40 ÷ 120 mm and l_emb_ = 240 ÷ 460 mm, are presented in Figure 20 and Figure 21, respectively. Corresponding f_b,ave-s_ distributions obtained from specimens after 28 days of concrete curing are displayed in Figure 22 and Figure 23. In view of the results obtained, the relationships f_b,ave-s_ determined for test specimens with l_emb_ = 240 ÷ 460 mm can be considered representative.

**Figure 24:**
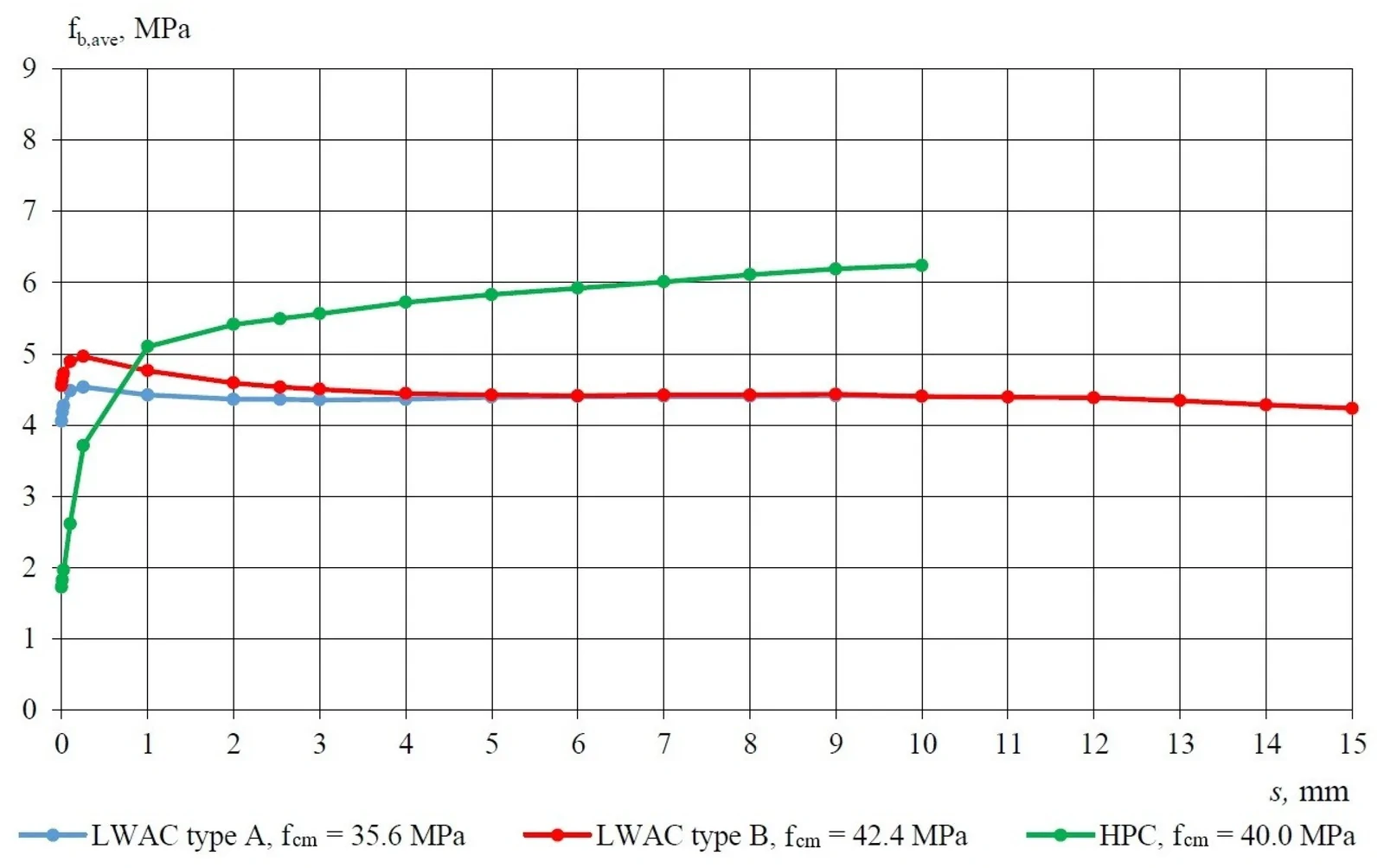
Average values of bond stress–slip relationship (*f_b,ave_* − *s*) for LWACs and HPC with concrete compressive strength ranging from 35.6 to 42.4 MPa to plain seven-wire steel strand of 15.7 mm diameter for l_emb_ = 240 ÷ 460 mm (240 ÷ 330 mm). Specimen cross-section of 160 × 160 mm.

**Figure 25:**
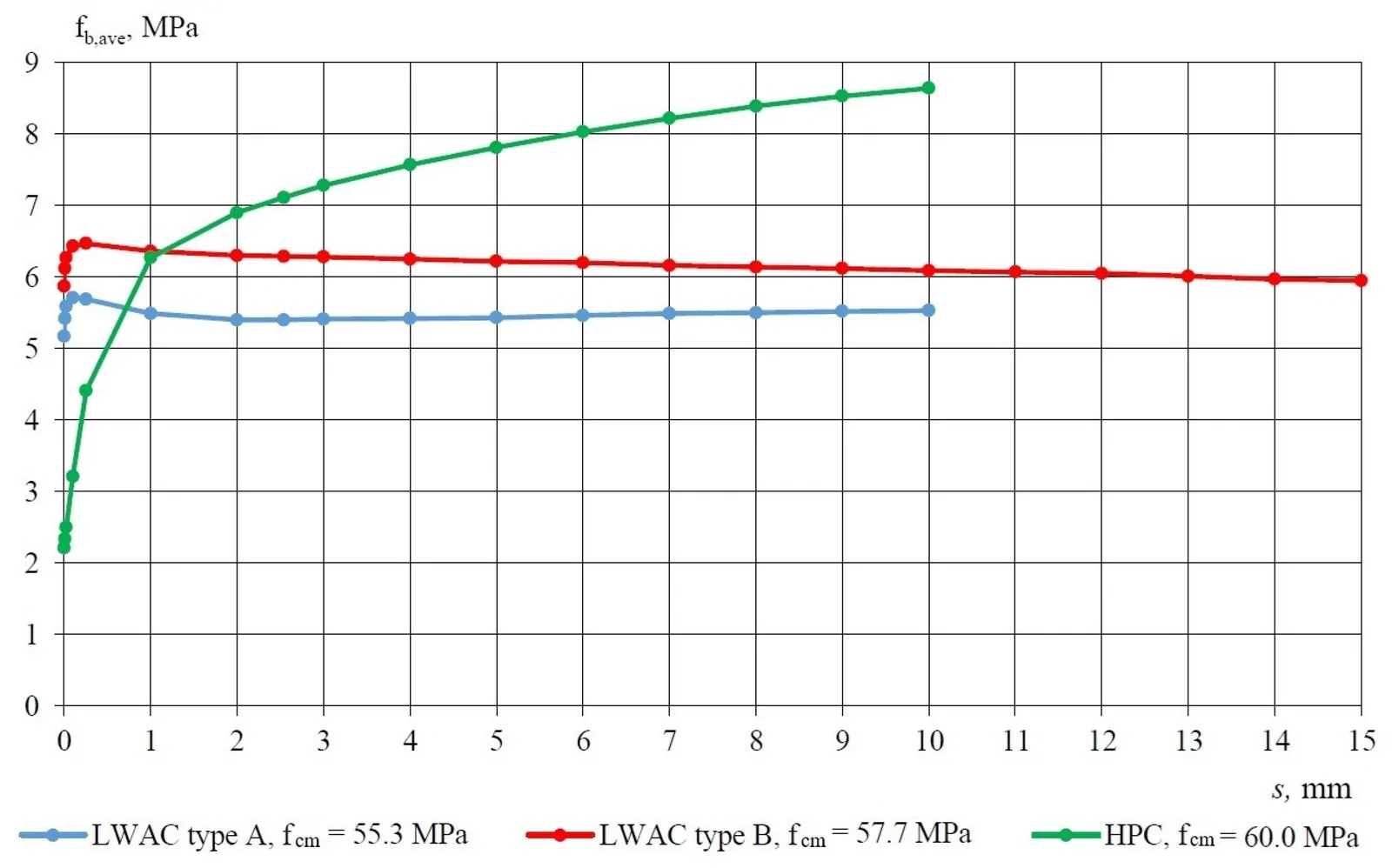
Average values of the bond stress–slip relationship (*f_b,ave_* − *s*) for LWACs and HPC with concrete compressive strength ranging from 55.3 to 60.0 MPa to plain seven-wire steel strand of 15.7 mm diameter for l_emb_ = 240 ÷ 460 mm (240 ÷ 330 mm). Specimen cross-section of 160 × 160 mm.

Considering the results of the bond stress–slip relationship for specimens with embedment lengths of 240, 330, and 460 mm for LWAC type B, the dependency of the relative bond stress f_b_/f_cm_ and f_b_/√f_cm_ was analyzed. The effect of the square root of LWAC compressive strength on the relative bond stress–slip relationship is detailed in Table 13. The results obtained from the analysis of test specimens made of type A concrete are presented in Table 14, Table 15 and Table 16. Figure 24 and Figure 25 present a comparison of the average bond stress–slip relationships corresponding to the compressive strengths of lightweight aggregate concretes (LWACs) and high-performance concrete (HPC) specimens that are composed of concretes with comparable compressive strengths. The graphs demonstrate that the adhesion stress for both types (A and B) of LWACs is considerably higher than that of HPC, with LWACs showing no further increase in bond stress. This finding suggests a lack of the aggregate interlocking effect. In contrast, HPC exhibits this interlocking effect, leading to a steady increase in bond stress.

Average values of the maximum force pushing the LWAC type A and type B specimens of the non-pretensioned steel strand are presented in Table 14. Additionally, Table 15 provides the average values of the maximum bond stress between the LWAC specimens and the non-pretensioned steel strand. Furthermore, Table 16 presents the average values of the maximum stress within the non-pretensioned steel strand. It can be seen that the average maximum strand stress values are registered in concrete specimens with compressive strengths of 42.4 MPa and 57.7 MPa for an embedment length of 460 mm. Table 17 illustrates the relationship between the real strand stress level and the ultimate strength ratio for the examined embedment lengths. The maximum ratios of strand stress to ultimate strength for an embedment length of 460 mm are 0.463 and 0.618 for concrete compressive strengths of 42.4 MPa and 57.7 MPa, respectively. It is important to note that all concrete specimens underwent testing with continuous slip data recording, and no cracks were detected on the external surfaces of the test specimens.

The concrete utilizing the artificial lightweight aggregate “Certyd” is characterized by a high adhesive bond strength, coupled with a notably low development of the interlocking mechanism. This characteristic is evident in the distinct behavior of the bond stress–slip relationship.

For research purposes, average values of the bond stress–slip relationship for lightweight aggregate concrete (LWAC) types A and B, along with high-performance concrete (HPC) in relation to plain seven-wire non-pretensioned steel strand of 15.7 mm, are presented in Table 18. The bond stress–slip relationship distributions for the examined concrete specimens, which possess a cross-section of 160 × 160 mm, are depicted in Figure 24 and Figure 25. The graphs reveal that the adhesion stress for LWAC with Certyd aggregate is significantly higher than that of high-performance concrete [24]. This observation indicates a lack of the aggregate interlocking effect. Conversely, in the case of HPC, this interlocking effect is present, resulting in a gradual increase in bond stress.

**Table 13:** Influence of the square root of the LWAC compressive strength on the relative bond stress–slip relationship for 15.7 mm steel strand.

s mm	15.7 mm Diameter Non-Pretensioned Steel Strand, Specimen Cross-Section 160 × 160 mm, f_b,ave_/√f_cm_												
	l_emb_ = 240 mm				l_emb_ = 330 mm				l_emb_ = 460 mm				Average
	f_cm_, MPa		Aver.	CoV %	f_cm_, MPa		Aver.	CoV %	f_cm_, MPa		Aver.	CoV %	
	42.4	57.7			42.4	57.7			42.4	57.7			
adhes.	0.689	0.821	0.755	8.78	0.756	0.823	0.789	4.21	0.650	0.674	0.662	1.82	0.735
0.01	0.716	0.842	0.779	8.08	0.764	0.832	0.798	4.27	0.654	0.742	0.698	6.31	0.758
0.0254	0.744	0.860	0.802	7.19	0.771	0.851	0.811	4.91	0.659	0.766	0.713	7.51	0.775
0.1	0.784	0.901	0.843	6.89	0.791	0.864	0.827	4.40	0.675	0.774	0.725	6.80	0.798
0.254	0.806	0.909	0.857	5.99	0.807	0.874	0.840	3.96	0.670	0.772	0.721	7.07	0.806
1	0.780	0.869	0.824	5.45	0.803	0.886	0.845	4.90	0.607	0.756	0.681	10.89	0.784
2	0.752	0.842	0.797	5.62	0.799	0.896	0.848	5.70	0.564	0.749	0.657	14.03	0.767
2.54	0.739	0.834	0.787	5.99	0.796	0.902	0.849	6.23	0.553	0.748	0.650	15.02	0.762
3	0.731	0.828	0.779	6.20	0.795	0.905	0.850	6.45	0.544	0.747	0.646	15.64	0.758
4	0.713	0.821	0.767	7.07	0.798	0.907	0.853	6.38	0.533	0.740	0.636	16.22	0.752
5	0.704	0.816	0.760	7.36	0.803	0.905	0.854	6.00	0.527	0.736	0.632	16.57	0.749
6	0.703	0.810	0.756	7.06	0.803	0.905	0.854	5.97	0.527	0.732	0.629	16.35	0.746
7	0.703	0.802	0.752	6.56	0.802	0.907	0.854	6.18	0.530	0.725	0.628	15.53	0.745
8	0.699	0.796	0.748	6.41	0.805	0.908	0.856	5.99	0.530	0.719	0.625	15.16	0.743
9	0.696	0.794	0.745	6.55	0.804	0.907	0.856	6.04	0.538	0.714	0.626	14.13	0.742
10	0.693	0.793	0.743	6.75	0.798	0.902	0.850	6.16	0.537	0.711	0.624	14.02	0.739
11	0.687	0.794	0.741	7.20	0.788	0.896	0.842	6.43	0.545	0.706	0.625	12.88	0.736
12	0.681	0.798	0.739	7.91	0.783	0.892	0.838	6.55	0.552	0.700	0.626	11.85	0.734
13	0.673	0.801	0.736	8.69	0.770	0.880	0.825	6.71	0.554	0.693	0.623	11.10	0.728
14	0.651	0.802	0.727	10.39	0.762	0.871	0.816	6.68	0.558	0.685	0.622	10.25	0.722
15	0.639	0.804	0.722	11.43	0.752	0.863	0.808	6.87	0.555	0.681	0.618	10.21	0.716

**Table 14:** Average values of the maximum force pushing the LWAC specimen off the non-pretensioned steel strand F_max,ave_, kN.

l_emb_ mm	LWAC Type A		LWAC Type B	
	f_cm_ = 35.6 MPa	f_cm_ = 55.3 MPa	f_cm_ = 42.4 MPa	f_cm_ = 57.7 MPa
	F_max,ave_, kN	F_max,ave_, kN	F_max,ave_, kN	F_max,ave_, kN
40	10.65	16.94	10.50	16.85
80	32.84	38.54	22.64	37.75
120	41.24	52.03	33.01	44.79
240	84.32	102.58	82.97	110.09
330	95.86	121.69	117.10	152.33
460	128.90	165.67	133.48	178.27

**Table 15:** Average values of the maximum bond stress of the LWAC specimen to the non-pretensioned steel strand f_bmax,ave_, MPa.

l_emb_ mm	LWAC Type A		LWAC Type B	
	f_cm_ = 35.6 MPa	f_cm_ = 55.3 MPa	f_cm_ = 42.4 MPa	f_cm_ = 57.7 MPa
	3 Days	28 Days	3 Days	28 Days
40	4.05	6.44	3.99	6.41
80	6.24	7.33	4.30	7.18
120	5.23	6.60	4.18	5.68
240	5.34	6.50	5.26	6.97
330	4.42	5.61	5.40	7.02
460	4.26	5.48	4.41	5.89

**Table 16:** Average values of the maximum stress in the non-pretensioned steel strand f_pmax,ave_, MPa.

l_emb_ mm	LWAC Type A		LWAC Type B	
	f_cm_ = 35.6 MPa	f_cm_ = 55.3 MPa	f_cm_ = 42.4 MPa	f_cm_ = 57.7 MPa
	3 Days	28 Days	3 Days	28 Days
40	71.00	112.93	69.54	111.59
80	218.93	256.93	149.93	250.00
120	274.93	346.87	218.61	296.62
240	562.13	683.87	549.47	729.07
330	639.07	811.27	775.50	1008.81
460	859.33	1104.47	883.97	1180.60

**Table 17:** Real strand stress level to ultimate strength ratio (*f_pmax,ave_*/*f_pk_*) for prestressing steel strand.

l_emb_ mm	LWAC Type A		LWAC Type B	
	f_cm_ = 35.6 MPa	f_cm_ = 55.3 MPa	f_cm_ = 42.4 MPa	f_cm_ = 57.7 MPa
	3 Days	28 Days	3 Days	28 Days
40	0.037	0.059	0.036	0.058
80	0.115	0.135	0.078	0.131
120	0.144	0.182	0.114	0.155
240	0.295	0.359	0.288	0.382
330	0.335	0.425	0.406	0.528
460	0.451	0.579	0.463	0.618

**Table 18:** Average values of bond stress–slip relationship for LWAC and HPC to plain seven-wire non-pretensioned steel strand of 15.7 mm. Specimen cross-section of 160 × 160 mm.

s mm	Average Values of Bond Stress–Slip Relationship to Plain Seven-Wire Non-Pretensioned Steel Strand of 15.7 mm					
	LWAC Type A		LWAC Type B		HPC	
	l_emb_ = 240 ÷ 460 mm		l_emb_ = 240 ÷ 460 mm		l_emb_ = 240 and 330 mm	
	f_cm_, MPa					
	35.6	55.3	42.4	57.7	40	60
adhesion	4.05	5.17	4.55	5.87	1.72	2.21
0.01	4.18	5.42	4.63	6.12	1.82	2.34
0.0254	4.27	5.59	4.72	6.27	1.96	2.50
0.1	4.48	5.71	4.89	6.43	2.61	3.21
0.254	4.53	5.69	4.96	6.47	3.71	4.41
1	4.42	5.49	4.76	6.36	5.10	6.27
2	4.36	5.40	4.59	6.30	5.41	6.90
2.54	4.36	5.40	4.53	6.29	5.49	7.11
3	4.35	5.41	4.50	6.28	5.56	7.28
4	4.36	5.42	4.44	6.25	5.72	7.57
5	4.39	5.43	4.42	6.22	5.83	7.81
6	4.40	5.46	4.41	6.20	5.92	8.03
7	4.40	5.49	4.42	6.16	6.01	8.22
8	4.40	5.50	4.42	6.14	6.11	8.39
9	4.41	5.52	4.43	6.12	6.19	8.53
10	4.40	5.53	4.40	6.09	6.24	8.64
11	-	-	4.39	6.07	-	-
12	-	-	4.38	6.05	-	-
13	-	-	4.34	6.01	-	-
14	-	-	4.28	5.97	-	-
15	-	-	4.23	5.95	-	-

## 6. Conclusions

Based on the results obtained from both experimental investigations and theoretical calculations, the following conclusions regarding the bond behavior of non-pretensioned steel strands in lightweight aggregate concrete have been established:

- Experimental studies on the relationship between bond stress and slip for a seven-wire non-prestressed steel strand embedded in lightweight concrete made with the artificial aggregate “Certyd” demonstrated that the interlocking mechanism does not influence the development of bond stress, regardless of the bond length of the test specimens. After overcoming the adhesive bond, there is an approximate 10% increase in bond stress, reaching a maximum value at a slip of 0.254 mm, followed by a slight decrease and stabilization of the residual bond stress at slips of up to 10 or 15 mm. More consistent results were obtained from specimens with bond lengths ranging from 240 to 460 mm. The coefficient of variation for the average bond stress ranges from a few to several percent. In the case of specimens with bond lengths of 40 to 120 mm, the coefficient of variation can reach as high as 40%.
- The adhesive bond in lightweight aggregate concrete containing sintered fly ash to the non-pretensioned steel strand with a diameter of 15.7 mm is not uniform. It varies with the embedment length and the compressive strength of the concrete. The average adhesive bond value f_ba,ave_ determined for specimens with embedment lengths l_emb_ = 240 to 460 mm made from type A concrete is 0.87·f_bmax,ave_ and 0.88·f_bmax,ave_ after 3 and 28 days of concrete curing, respectively. For specimens with l_emb_ = 240 to 460 mm made from type B concrete, the average adhesive bond value is f_ba,ave_ = 0.90·f_bmax,ave_ and 0.88·f_bmax,ave_ after 3 and 28 days of curing, respectively.
- The average maximum bond strength was found at a steel strand slip of less than 0.254 mm for both LWAC type A and LWAC type B. When the slip exceeds 1 mm, a stabilization of the average bond stress to the strand at 15.7 mm is observed for both concrete types.
- Experimental investigations carried out at the Cracow University of Technology validated that, to accurately assess the bond strength to seven-wire strands, test specimens should be prepared with a bond length that is at least equal to the strand lay length (pitch). In the context of the authors’ experiments, the results for l_emb_ values ranging from 240 to 460 mm are considered reliable.
- The experimental data revealed that bond strength increases in proportion to the square root of the compressive strength. For LWAC type A, the adhesion bond is defined by the equation f_ba_ = 0.681·√f_cm_ for concrete compressive strengths ranging from 35 to 55 MPa. For LWAC type B, the adhesion bond can be expressed as f_ba_ = 0.735·√f_cm_ for concrete compressive strengths between 42 and 57 MPa. These average bond stress values were derived from experimental results for concrete specimens with embedment lengths of 240, 330, and 460 mm.
- The average bond strength for slip values from 1 to 10 mm between lightweight aggregate concrete and non-pretensioned seven-wire steel strand of 15.7 mm diameter can be estimated using the formulas: f_b,ave_ = 0.731·√f_cm_ for LWAC type A (concrete compressive strength 35 to 55 MPa) and f_b,ave_ = 0.753·√f_cm_ for LWAC type B (concrete compressive strength 42 to 57 MPa).
- After a curing period of 3 and 28 days for concrete type B, the average bond stress values obtained were greater than those observed in concrete type A produced with CEM I 52.5R cement. The average bond stress for strand slip values between 1 and 10 mm was 4.48 MPa, with f_cm_ = 42.4 MPa (4.39 MPa for concrete specimens using CEM I 52.5R, f_cm_ = 35.3 MPa) and 6.22 MPa, with f_cm_ = 57.7 MPa (5.46 MPa for concrete specimens using CEM I 52.5R, f_cm_ = 55.3 MPa) after curing periods of 3 and 28 days, respectively. It is important to mention that for normal-weight concretes, the ACI standard for determining transmission length and anchorage length restricts the bond stress value to 3.0 MPa.
- The increased bond stress values observed in type B concrete over type A concrete after 28 days of maturation, while both exhibit comparable compressive strengths, are attributed to the presence of siliceous fly ash in the cement that seals the structure of the concrete.
- The bond strength of type B concrete, assessed on test specimens with l_emb_ = 240 ÷ 460 mm, along with the observed bond loss mechanism between the concrete and the seven-wire steel strand, shows similarities to the f_b_ values and the performance of elements constructed from type A concrete. The conducted test confirmed that Certyd LWAC made in a concrete mixture with cement type CEM II/A-V 52.5R-NA is appropriate for pretensioned concrete members, as well as cement type CEM I 52.5R. Additionally, it allows for the reduction in the carbon footprint and enhances the workability of the concrete mixture.
- Previous studies conducted on elements made of concrete type A included tests of bond strength, with non-pretensioned and pretensioned steel strands. Furthermore, those studies were performed on transmission length and anchorage length in beam elements [18]. The second phase of the current research program on elements made of concrete type B will involve testing of specimens reinforced with pretensioned steel strands.

## Figures and Tables

**Table 1:** Mix design for light-weight aggregate concrete [17].

Components		Quantities, kg/m^3^
Rapid-hardening Portland Cement type A/type B		419
River Sand (0–2 mm)		703
Certyd aggregate (4–12 mm)		802
Water		209
Additives	BV 18, high-performance superplasticiser, BASF	3.8
	SKY 686, h-r water-reducing superplasticiser, BASF	3.8
*w*/*c* ratio		0.50

**Table 2:** LWAC type A specimens with a cross-section of 160 × 160 mm reinforced with a non-pretensioned steel strand of 15.7 mm diameter [17].

l_emb_ mm	Number of the Specimen Tested	
	f_cm_, MPa	
	35.6	55.3
40	5	5
80	5	5
120	5	4
240	5	5
330	5	5
460	5	5
n	30	29
total	59	

**Table 3:** LWAC type B specimens with a cross-section of 160 × 160 mm reinforced with non-pretensioned steel strands of 15.7 mm diameter.

l_emb_ mm	Number of the Specimen Tested	
	f_cm_, MPa	
	42.4	57.7
40	3	3
80	3	3
120	3	3
240	3	3
330	3	3
460	3	3
n	18	18
total	36	

**Table 4:** Nominal properties of prestressing steel strand used with concrete type A specimens [17].

Geometry			Material Properties			
Strand diameter mm	Steel area mm^2^	Lay length mm	Ultimate strength *f_p_*, MPa	Breaking strength *F_pmax_*, kN	Yield strength *F_p_*_01_, kN	Modulus of elast. *E_p_*, MPa
15.7	150	249	1906.8	286.03	251.80	190,110

**Table 5:** Nominal properties of prestressing steel strand used with concrete type B specimens.

Geometry			Material Properties			
Strand diameter mm	Steel area mm^2^	Lay length mm	Ultimate strength *f_p_*, MPa	Breaking strength *F_pmax_*, kN	Yield strength *F_p_*_01_, kN	Modulus of elast. *E_p_*, MPa
15.7	151	252	1911.0	288.85	264.50	192,700

**Table 6:** Concrete mechanical properties.

Concrete Age, Days	Concrete Type A			Concrete Type B	
	f_cm_, MPa	f_ct,spl_, MPa	E_c_, MPa	f_c_, MPa	E_c_, MPa
3	35.65	2.97	21,560	42.44	23,530
7	45.18	3.25	22,315	-	-
28	55.28	3.63	24,200	57.72	26,140
90	-	-	-	67.63	27,670

**Table 7:** Average values of force pushing the LWAC type B specimen off the non-pretensioned steel strand (f_cm_ = 42.4 MPa, 3 days).

s mm	l_emb_, mm											
	40		80		120		240		330		460	
	F_ave_, kN	CoV %	F_ave_, kN	CoV %	F_ave_, kN	CoV %	F_ave_, kN	CoV %	F_ave_, kN	CoV %	F_ave_, kN	CoV %
adhesion	7.99	16.43	19.45	9.38	31.20	8.75	70.82	15.11	106.88	1.84	128.19	8.70
0.01	9.40	2.26	20.78	3.16	32.22	9.13	73.66	11.57	108.00	1.69	128.92	7.87
0.0254	9.90	7.00	21.34	4.58	32.45	8.50	76.54	9.68	108.97	1.90	129.88	6.85
0.1	10.04	17.58	21.77	9.10	31.05	17.85	80.67	11.07	111.82	2.39	133.15	2.63
0.254	9.75	22.15	21.10	13.10	28.42	24.38	82.88	11.72	114.11	2.44	132.06	1.87
1	7.90	22.75	18.85	21.88	24.09	30.16	80.13	11.26	113.59	0.53	119.68	1.07
2	7.30	20.75	18.30	27.08	23.04	32.31	77.32	10.34	112.99	0.70	111.31	0.62
2.54	7.10	20.56	18.41	29.12	22.99	34.06	76.04	9.13	112.58	0.85	108.88	0.48
3	6.92	21.63	18.53	30.35	23.02	33.83	75.18	8.33	112.43	0.85	107.33	0.76
4	6.74	17.31	18.48	33.66	23.35	35.02	73.28	7.66	112.88	1.08	105.09	0.86
5	6.64	13.78	18.69	35.28	23.67	34.60	72.42	7.36	113.45	1.59	103.85	0.75
6	6.40	12.18	18.60	37.94	24.03	35.64	72.30	7.18	113.53	2.30	103.70	0.47
7	5.96	13.55	18.73	40.63	24.48	36.81	72.23	7.02	113.29	2.80	104.48	1.90
8	5.82	11.03	18.90	41.28	24.75	37.43	71.94	7.43	113.80	3.95	104.44	2.99
9	5.78	9.10	18.70	41.05	24.88	36.67	71.60	8.55	113.67	4.88	105.92	5.08
10	5.52	8.98	18.36	39.83	24.58	35.97	71.26	9.25	112.74	5.76	105.70	5.12
11	5.34	7.74	18.05	40.43	24.25	34.96	70.69	10.16	111.40	5.83	107.44	6.76
12	5.21	6.39	17.55	40.33	24.20	35.00	70.00	10.94	110.67	6.46	108.81	7.69
13	5.17	4.33	17.27	39.69	24.25	33.61	69.14	10.79	108.77	5.37	109.21	7.52
14	5.19	2.62	17.26	39.10	24.18	31.09	66.97	8.98	107.70	5.11	110.00	7.05
15	5.20	0.77	17.12	38.55	24.10	28.39	65.71	9.02	106.29	4.87	109.43	6.36
F_max,ave_	10.50	15.84	22.64	9.25	33.01	10.00	82.97	11.72	117.10	2.34	133.48	2.47

**Table 8:** Average values of force pushing the LWAC type B specimen off the non-pretensioned steel strand (f_cm_ = 57.7 MPa, 28 days).

s mm	l_emb_, mm											
	40		80		120		240		330		460	
	F_ave_, kN	CoV %	F_ave_, kN	CoV %	F_ave_, kN	CoV %	F_ave_, kN	CoV %	F_ave_, kN	CoV %	F_ave_, kN	CoV %
adhesion	14.07	10.69	30.62	10.17	38.00	17.02	98.49	3.78	135.61	0.98	155.04	4.68
0.01	15.92	13.19	33.87	8.58	40.23	13.85	101.01	2.40	137.19	0.20	170.59	4.05
0.0254	16.63	11.94	36.19	2.10	40.59	13.58	103.10	1.11	140.21	2.41	176.08	4.43
0.1	15.51	12.79	36.97	2.26	39.65	14.76	108.01	3.80	142.41	2.83	177.95	4.01
0.254	15.11	10.23	36.50	4.14	38.07	15.99	108.97	5.20	144.04	3.36	177.46	4.05
1	14.23	3.57	33.38	5.63	35.53	13.48	104.23	7.19	146.12	4.94	173.69	4.73
2	14.38	5.38	33.18	8.09	35.48	12.78	100.91	7.46	147.68	6.91	172.20	6.12
2.54	14.50	6.78	33.15	10.03	35.76	14.17	99.98	7.18	148.74	7.63	171.87	6.60
3	14.35	9.37	33.12	10.98	36.47	14.43	99.26	7.00	149.21	8.00	171.60	7.04
4	14.72	8.68	33.60	12.68	38.22	16.40	98.47	5.74	149.59	8.19	170.01	6.82
5	14.18	12.71	33.65	12.54	39.73	17.19	97.88	4.26	149.20	7.31	169.21	5.82
6	13.84	14.73	33.44	9.06	41.20	15.90	97.11	3.11	149.20	6.65	168.19	4.10
7	12.83	21.44	33.60	7.12	41.93	16.78	96.07	3.31	149.53	5.91	166.67	3.43
8	12.91	23.63	33.74	8.04	42.35	16.12	95.39	3.49	149.64	4.90	165.31	2.63
9	13.01	25.42	34.12	8.63	42.68	14.23	95.19	4.07	149.61	4.17	164.17	2.53
10	13.05	26.12	33.61	6.92	42.60	15.29	95.13	4.84	148.74	3.90	163.48	2.14
11	12.65	28.49	33.29	5.98	42.75	15.33	95.23	5.42	147.76	3.94	162.34	1.05
12	12.24	27.43	32.85	6.61	43.44	14.43	95.66	6.24	147.15	3.82	161.02	1.06
13	12.00	25.83	32.90	6.11	43.64	14.15	95.98	7.07	145.11	4.42	159.17	1.69
14	11.49	21.94	32.98	6.90	43.34	14.89	96.20	7.60	143.56	4.85	157.60	2.36
15	10.41	7.79	32.80	7.59	43.31	16.00	96.41	8.41	142.24	5.02	156.63	2.60
F_max, ave_	16.85	10.89	37.75	2.55	44.79	12.76	110.09	4.21	152.33	5.47	178.27	4.02

**Table 9:** Bond stress average values of LWAC type B specimens to the non-pretensioned steel strand (f_cm_ = 42.4 MPa, 3 days).

s mm	l_emb_, mm											
	40		80		120		240		330		460	
	f_b.ave_, MPa	CoV %	f_b.ave_, MPa	CoV %	f_b.ave_, MPa	CoV %	f_b.ave_, MPa	CoV %	f_b.ave_, MPa	CoV %	f_b.ave_, MPa	CoV %
adhesion	3.04	16.43	3.70	9.38	3.95	8.75	4.49	15.11	4.92	1.84	4.24	8.70
0.01	3.57	2.26	3.95	3.16	4.08	9.13	4.67	11.57	4.98	1.69	4.26	7.87
0.0254	3.76	7.00	4.06	4.58	4.11	8.50	4.85	9.68	5.02	1.90	4.29	6.85
0.1	3.82	17.58	4.14	9.10	3.93	17.85	5.11	11.07	5.15	2.39	4.40	2.63
0.254	3.71	22.15	4.01	13.10	3.60	24.38	5.25	11.72	5.26	2.44	4.37	1.87
1	3.00	22.75	3.58	21.88	3.05	30.16	5.08	11.26	5.23	0.53	3.96	1.07
2	2.78	20.75	3.48	27.08	2.92	32.31	4.90	10.34	5.21	0.70	3.68	0.62
2.54	2.70	20.56	3.50	29.12	2.91	34.06	4.82	9.13	5.19	0.85	3.60	0.48
3	2.63	21.63	3.52	30.35	2.92	33.83	4.76	8.33	5.18	0.85	3.55	0.76
4	2.56	17.31	3.51	33.66	2.96	35.02	4.64	7.66	5.20	1.08	3.47	0.86
5	2.53	13.78	3.55	35.28	3.00	34.60	4.59	7.36	5.23	1.59	3.43	0.75
6	2.43	12.18	3.53	37.94	3.05	35.64	4.58	7.18	5.23	2.30	3.43	0.47
7	2.26	13.55	3.56	40.63	3.10	36.81	4.58	7.02	5.22	2.80	3.45	1.90
8	2.21	11.03	3.59	41.28	3.14	37.43	4.56	7.43	5.24	3.95	3.45	2.99
9	2.20	9.10	3.55	41.05	3.15	36.67	4.54	8.55	5.24	4.88	3.50	5.08
10	2.10	8.98	3.49	39.83	3.11	35.97	4.51	9.25	5.19	5.76	3.49	5.12
11	2.03	7.74	3.43	40.43	3.07	34.96	4.48	10.16	5.13	5.83	3.55	6.76
12	1.98	6.39	3.34	40.33	3.07	35.00	4.44	10.94	5.10	6.46	3.60	7.69
13	1.96	4.33	3.28	39.69	3.07	33.61	4.38	10.79	5.01	5.37	3.61	7.52
14	1.97	2.62	3.28	39.10	3.06	31.09	4.24	8.98	4.96	5.11	3.64	7.05
15	1.98	0.77	3.25	38.55	3.05	28.39	4.16	9.02	4.90	4.87	3.62	6.36
*f_b max,ave_*	3.99	15.84	4.30	9.25	4.18	10.00	5.26	11.72	5.40	2.34	4.41	2.47
f_b.ave_ s = 1 ÷ 10 mm	2.49	11.25	3.53	1.03	3.03	3.01	4.69	3.85	5.21	0.41	3.55	4.45
f_b.ave_ s = 1 ÷ 15 mm	2.33	14.31	3.47	3.29	3.04	2.52	4.58	5.09	5.15	2.07	3.56	3.73

**Table 10:** Bond stress average values of LWAC type B specimen to the non-pretensioned steel strand (f_cm_ = 57.7 MPa, 28 days).

s mm	l_emb_, mm											
	40		80		120		240		330		460	
	f_b.ave_, MPa	CoV %	f_b.ave_, MPa	CoV %	f_b.ave_, MPa	CoV %	f_b.ave_, MPa	CoV %	f_b.ave_, MPa	CoV %	f_b.ave_, MPa	CoV %
adhesion	5.35	10.69	5.82	10.17	4.82	17.02	6.24	3.78	6.25	0.98	5.12	4.68
0.01	6.05	13.19	6.44	8.58	5.10	13.85	6.40	2.40	6.32	0.20	5.64	4.05
0.0254	6.32	11.94	6.88	2.10	5.14	13.58	6.53	1.11	6.46	2.41	5.82	4.43
0.1	5.89	12.79	7.03	2.26	5.02	14.76	6.84	3.80	6.56	2.83	5.88	4.01
0.254	5.75	10.23	6.94	4.14	4.82	15.99	6.90	5.20	6.64	3.36	5.87	4.05
1	5.41	3.57	6.34	5.63	4.50	13.48	6.60	7.19	6.73	4.94	5.74	4.73
2	5.47	5.38	6.31	8.09	4.50	12.78	6.39	7.46	6.81	6.91	5.69	6.12
2.54	5.51	6.78	6.30	10.03	4.53	14.17	6.33	7.18	6.85	7.63	5.68	6.60
3	5.46	9.37	6.29	10.98	4.62	14.43	6.29	7.00	6.88	8.00	5.67	7.04
4	5.60	8.68	6.39	12.68	4.84	16.40	6.24	5.74	6.89	8.19	5.62	6.82
5	5.39	12.71	6.40	12.54	5.03	17.19	6.20	4.26	6.87	7.31	5.59	5.82
6	5.26	14.73	6.36	9.06	5.22	15.90	6.15	3.11	6.88	6.65	5.56	4.10
7	4.88	21.44	6.39	7.12	5.31	16.78	6.09	3.31	6.89	5.91	5.51	3.43
8	4.91	23.63	6.41	8.04	5.37	16.12	6.04	3.49	6.90	4.90	5.46	2.63
9	4.95	25.42	6.49	8.63	5.41	14.23	6.03	4.07	6.89	4.17	5.43	2.53
10	4.96	26.12	6.39	6.92	5.40	15.29	6.03	4.84	6.85	3.90	5.40	2.14
11	4.81	28.49	6.33	5.98	5.42	15.33	6.03	5.42	6.81	3.94	5.37	1.05
12	4.65	27.43	6.24	6.61	5.50	14.43	6.06	6.24	6.78	3.82	5.32	1.06
13	4.56	25.83	6.25	6.11	5.53	14.15	6.08	7.07	6.69	4.42	5.26	1.69
14	4.37	21.94	6.27	6.90	5.49	14.89	6.10	7.60	6.62	4.85	5.21	2.36
15	3.96	7.79	6.23	7.59	5.49	16.00	6.11	8.41	6.55	5.02	5.18	2.60
*f_b max,ave_*	6.41	10.89	7.18	2.55	5.68	12.76	6.97	4.21	7.02	5.47	5.89	4.02
f_b.ave_ s = 1 ÷ 10 mm	5.25	5.23	6.37	0.92	4.98	7.77	6.22	2.87	6.86	0.73	5.58	2.06
f_b.ave_ s = 1 ÷ 15 mm	5.01	9.36	6.34	1.14	5.14	7.79	6.17	2.61	6.81	1.56	5.48	3.28

## Data Availability

The original contributions presented in this study are included in the article. Further inquiries can be directed to the corresponding author.
